# OMICs, Epigenetics, and Genome Editing Techniques for Food and Nutritional Security

**DOI:** 10.3390/plants10071423

**Published:** 2021-07-12

**Authors:** Yuri V. Gogolev, Sunny Ahmar, Bala Ani Akpinar, Hikmet Budak, Alexey S. Kiryushkin, Vladimir Y. Gorshkov, Goetz Hensel, Kirill N. Demchenko, Igor Kovalchuk, Freddy Mora-Poblete, Tugdem Muslu, Ivan D. Tsers, Narendra Singh Yadav, Viktor Korzun

**Affiliations:** 1 Federal Research Center Kazan Scientific Center of Russian Academy of Sciences, Kazan Institute of Biochemistry and Biophysics, 420111 Kazan, Russia; gvy84@mail.ru; 2 Federal Research Center Kazan Scientific Center of Russian Academy of Sciences, Laboratory of Plant Infectious Diseases, 420111 Kazan, Russia; ivantsers@gmail.com; 3 Institute of Biological Sciences, University of Talca, 1 Poniente 1141, Talca 3460000, Chile; sunnyahmar13@gmail.com (S.A.); morapoblete@gmail.com (F.M.-P.); 4 Montana BioAg Inc., Missoula, MT 59802, USA; aniakpinar@gmail.com (B.A.A.); hikmet.budak@icloud.com (H.B.); 5 Laboratory of Cellular and Molecular Mechanisms of Plant Development, Komarov Botanical Institute of the Russian Academy of Sciences, 197376 Saint Petersburg, Russia; akiryushkin@binran.ru (A.S.K.); demchenko@binran.ru (K.N.D.); 6 Centre for Plant Genome Engineering, Institute of Plant Biochemistry, Heinrich-Heine-University, 40225 Dusseldorf, Germany; goetz.hensel@hhu.de; 7 Centre of the Region Haná for Biotechnological and Agricultural Research, Czech Advanced Technology and Research Institute, Palacký University Olomouc, 78371 Olomouc, Czech Republic; 8 Department of Biological Sciences, University of Lethbridge, Lethbridge, AB T1K 3M4, Canada; igor.kovalchuk@uleth.ca (I.K.); nsyadava2004@gmail.com (N.S.Y.); 9 Faculty of Engineering and Natural Sciences, Sabanci University, 34956 Istanbul, Turkey; tugdem@sabanciuniv.edu; 10 KWS SAAT SE & Co. KGaA, Grimsehlstr. 31, 37555 Einbeck, Germany

**Keywords:** epigenetics, epigenomics, genome sequencing, genomic prediction, omics, plant microbiome, site-directed mutagenesis, transcriptome

## Abstract

The incredible success of crop breeding and agricultural innovation in the last century greatly contributed to the Green Revolution, which significantly increased yields and ensures food security, despite the population explosion. However, new challenges such as rapid climate change, deteriorating soil, and the accumulation of pollutants require much faster responses and more effective solutions that cannot be achieved through traditional breeding. Further prospects for increasing the efficiency of agriculture are undoubtedly associated with the inclusion in the breeding strategy of new knowledge obtained using high-throughput technologies and new tools in the future to ensure the design of new plant genomes and predict the desired phenotype. This article provides an overview of the current state of research in these areas, as well as the study of soil and plant microbiomes, and the prospective use of their potential in a new field of microbiome engineering. In terms of genomic and phenomic predictions, we also propose an integrated approach that combines high-density genotyping and high-throughput phenotyping techniques, which can improve the prediction accuracy of quantitative traits in crop species.

## 1. Introduction

The adequate supply of food providing calories and nutrients is essential for human survival. It has been estimated that the human population of approximately 800 million people worldwide is facing an acute shortage of food, and around two billion are facing nutrient deficiency [1]. Plant breeding and other agricultural technologies have contributed considerably to hunger reduction during the last few decades. However, crop improvement through the conventional breeding approaches is time-consuming and lacks the ability to deal with the global food requirements. Therefore, current research efforts in crop improvement target OMIC technologies (a field of study in biological sciences that ends with -omics, e.g., genomics, phenomics, proteomics, etc.) to improve the efficiency and accuracy of conventional breeding. The amount of crop improvement programs that use the principles of OMIC-assisted breeding has increased considerably in recent years. Moreover, the exploitation of the advantages of the high-throughput techniques could positively impact the genetic improvement of crops based on OMIC-assisted breeding.

## 2. Genomics

### 2.1. Plant Genome Sequencing and Annotation

The mystery of the deoxyribonucleic acid (DNA) molecule structure was unraveled in 1953 [2], but reading and sequencing DNA using first-generation technologies came after almost a quarter of a century. Insulin was the first biological molecule to be sequenced. This happened thanks to the work of Frederick Sanger and Hans Tuppy [3,4], who combined hydrolysis of the peptide molecules, the use of colored terminal amino acid labels, and chromatography. Importantly, they demonstrated conclusively for the first time that proteins had a defined sequence. Studies in protein sequencing in the 1950s paved the way for nucleic acid sequencing, which initially seemed quite challenging compared to protein sequencing [5], and 1965 marked the first time a nucleic acid was sequenced [6]. The challenges that hampered sequencing efforts on DNA molecules, such as the relatively larger sizes of these molecules and the lack of suitable enzymes to break down the DNA into smaller fragments, have been overcome in years.

Following a switch from the hydrolysis-based approaches to synthesis-based approaches, in 1975, Sanger and Coulson developed a ”plus and minus” method for sequencing of single-stranded DNA molecules using DNA polymerases [7]. In 1977, two groundbreaking methods, the Maxam–Gilbert method [8] and the Sanger chain termination method, were published describing the rapid sequencing of long DNA fragments [9]. These two methods are today known as first-generation sequencing methods. Specifically, the chain termination method introduced the use of 2′-3′ dideoxynucleotides (ddNTPs) in a small ratio to 2′ deoxynucleotides. The lack of the 3′ hydroxyl group terminated the chain elongation at specific locations for each of the four ddNTPs, yielding different-sized fragments. The products were then separated by their sizes using electrophoresis, and the sequence was deduced [9]. In later years, this method was improved and was highly automated through the use of fluorescently-labeled nucleotides instead of radiolabeled nucleotides and the inclusion of real-time detection of laser excitation of DNA sequences in thin capillaries where fragments were separated by size [10].

The Sanger method remained the dominant technique for 30 years until the emergence of next-generation sequencing (NGS) in 2005. NGS technologies have revolutionized nucleic acid sequencing by increasing the output and decreasing the cost dramatically [11]. Sanger sequencing is low cost for small sequences, and sample preparation is relatively simple compared to next-generation sequencing, but low throughput makes Sanger sequencing an option not suitable for sequencing the entire genomes of many species. Contrarily, NGS platforms are capable of parallel sequencing and provide the opportunity of metagenomic studies, whole-exome sequencing, targeted sequencing, high throughput RNA sequencing, studies of gene expression changes, and most importantly whole-genome sequencing. All NGS platforms require a library preparation step where the long strands of nucleic acids are fragmented, size-sorted, and adapters ligated to the ends of the fragments [5]. The library than can be sequenced from one end (single-end) or both ends (paired-end) [12]. Read length, throughput, read accuracy, read depth, and cost per base are the varying properties of each NGS platform [13].

Third-generation sequencing (TGS) was introduced in 2011 by Pacific BioSciences (PacBio, CA, USA) and Oxford Nanopore Technologies (ONT, Oxford, UK). The distinctive properties of TGS platforms from other platforms are single-molecule sequencing (SMS), elimination of PCR amplification, and real-time sequencing of individual nucleic acid molecules [14]. TGS enhanced the continuity, completeness, and correctness of genome sequences, which enabled high-quality de novo assemblies [15] with the capability of sequencing long-reads. SMS-real time (SMRT) of Pacific Biosciences [16] and Nanopore sequencing of Oxford Nanopore Technologies (ONT) are two approaches with reading lengths up to 200 kb and 2 Mb, respectively, and these two approaches are widely adopted in plant genomic studies as they provide higher continuity, fewer gaps, and less errors [17]. The long read length, high consensus accuracy, and low biased G-C content of TGS platforms are characteristics of TGS with advantages over first- and next-generation of sequencing [18]. With the sheer amount of high-throughput data generated by the NGS and TGS platforms, the current challenge is not to generate the sequence data but to analyze and interpret them in a biologically meaningful way. This typically require the use of vast computational sources and in some cases highly-specialized software taking into account the source and type of the sequence data.

As the most comprehensive view of a genome, whole-genome sequencing (WGS) is replacing genetic marker studies and partial sequencing of exonic regions, making WGS a game-changer in the field of genetic analysis [19,20], converting conservation genetics studies to conservation genomics. The advances in high-throughput sequencing platforms accelerated WGS studies in plants, where almost 400 plant species now have at least one genome published [21]. Plant genomes vary significantly in size, ranging from tens of megabases to more than one hundred gigabases, and pose significant challenges to genome sequencing and subsequent assembly of the sequences generated. Polyploidy is widespread among plants; in fact, even extant diploid species are often considered as paleopolyploids [22]. Polyploidy can arise from whole-genome duplications (WGDs), as in the case of autopolyploids, or from hybridizations followed by WGDs, as in allopolyploids. The allopolyploid bread wheat genome, for example, is composed of three sub-genomes and can contain as many as six copies of a gene [23]. Distinguishing homeologous or paralogous copies of genes that can retain high sequence homology can be extremely difficult, particularly with short-read sequencing data. Heterozygosity is also high among plant genomes, as many species are self-incompatible [24]. Further complicating the assembly of high-throughput sequences is the high content of repetitive sequences of several plant genomes. Transposon activity contributes to genome expansion in plants, and transposable elements can reach up to 80% of the genome [25,26]. Extremely long-reads from TGS platforms such as PacBio [27], or the combined use of extensive mapping data with high-throughput, high-depth sequencing data [23] can aid in resolving the complex architecture of plant genomes, as demonstrated in the notoriously complex bread wheat genome.

Sequence databases and genome browsers such as GenBank, Ensembl, and UCSS Genome Browser emerged with the development of sequencing technology through the 21st century. The number of sequences found in GenBank when established in 1982 was about half a million bases, and this number increases by 18 times each year, and by the end of 2020, it included more than 723 billion bases [5,28]. With the availability of a vast amount and variety of sequencing platforms, the challenge is sequencing and the accurate interpretation and analysis of the data [12]. Consequently, while the availability of a genome assembly is valuable, the ultimate resource for a plant species that can be of immediate use to the community is an annotated genome assembly. Structural annotation of a genome comprises the genetic elements, including genes, non-coding regions, and repetitive elements on the genome sequence. Specific software and guidelines exist to identify different genomic elements [24,29]; however, plants with complex genomes can often benefit from specialized pipelines capable of automating annotation to a high extent [30]. The availability of annotated sequences in closely related species can also aid in determining genomic features along a genome. Large-scale synteny, the conservation of the sequence and order of genes, is prominent in species with shared evolutionary histories and has been used successfully to annotate mostly grass genomes. On the other hand, functional annotation can be more challenging and requires experimental evidence, considering large gene families with moderate sequence similarities but divergent roles typically present in plant genomes [24].

The advances in NGS and long-read sequencing technologies facilitated the complete assembly of the genomes of many plant species [31]. Availability of genome sequences of multiple individuals of the same species revealed a high degree of variation among plant genomes and raised concerns about the sufficiency of a single reference genome [32]. The pangenome, first described by Tettelin et al. (2005), can be defined as the entire genomic repertoire of an organism and involves the genome sequences of multiple individuals of the same species [33]. For a complete functional annotation of genes and genomes, comprehension of the structural variations among genomes and their link to phenotype is needed [32]. Even though the pangenome studies are in the infancy period and the assembly of pangenomes is still challenging, the rapidly changing sequencing platforms and tools will increase the availability of pangenomes for a large number of diverse species providing a deeper understanding of plant genomics [34].

First and foremost, a whole genome sequence enables the discovery of genetic elements, including protein-coding genes and other non-coding regulatory elements, and facilitates the isolation and characterization of regions of interest within a genome. While the ability to define genomic elements regulating important agronomic traits is most certainly useful in breeding, genome sequencing has more to offer. As the NGS platforms become more accessible, it is now possible to predict thousands to millions of sequence-based molecular markers, such as Single Nucleotide Polymorphisms (SNPs) and Insertion-Site-Based Polymorphisms (ISBPs), which, once validated, can contribute to marker-assisted selection, where molecular markers linked to desired traits are selected when breeding for better varieties [35]. Even in the absence of a reference genome sequence, effective strategies, such as RAD-seq, can generate useful sequence data on fragmented, barcoded, and pooled DNA samples that can identify SNPs [36]. Similarly, Genotyping-by-sequencing (GBS) is a powerful technique that uses reduced representation libraries by restriction–fragmentation to identify SNPs that can later be used for genotyping breeding populations [37]. Despite the challenges by genotype x environment interactions, effective genotyping and phenotyping based on molecular markers obtained with NGS technologies can be combined with statistical models to predict the performance of a target genotype in genomic selection approaches [36]. Finally, the availability of genome sequences from a number of key species can reveal evolutionary histories that underlie diversification of crop plants that can shape breeding strategies for future demands [38]. It is important to note that while genome sequencing can greatly benefit breeding programs, such approaches usually demand technical expertise and robust computational power.

### 2.2. Plant Microbiome Analysis by Metagenomics

#### 2.2.1. Metagenomic Approaches

Microorganisms colonizing the rhizosphere and the endophytic compartment contribute to plants’ growth, fitness, and productivity [39]. Similar to other eukaryotes, plants can be viewed as “super-organisms” possessing a distinct microbiome and revealing close symbiotic relationships with their associated microorganisms [39]. A feature of plants is that they obtain the main pool of interacting microflora from the soil, an unprecedentedly rich source of microbial diversity on Earth [40,41]. In the middle of the last century, Vinogradsky’s direct observations showed that only a tiny part of this diversity is made up of cultivated microorganisms [42]. However, it took more than half a century before multi-omics-based analysis emerged as an effective tool for studying uncultivated “dark matter”. Today, metagenomic analysis is the main tool.

Metagenomics refers to the study of the collective genome of microorganisms from an environmental sample. The first results in this direction were achieved using a molecular hybridization platform implemented as a high-density 16S rRNA gene oligonucleotide microarray, referred to as the PhyloChip [39,43,44]. In addition, two central next-generation sequencing (NGS)-based strategies have been developed for microbial genome analysis: ribosomal DNA (rDNA) amplicon sequencing [45,46,47,48], and whole metagenome shotgun sequencing (shotgun metagenomics) [46,48]. In the first approach, the variable region of the 16S rRNA gene V3 V4 is most often used as a phylogenetic marker for the identification of bacteria and archaea [49]; amplification of the internal transcribed spacer (ITS) regions of rDNA, in particular ITS2 between the 5.8S and 28S genes, followed by sequencing, is widely used for the detection and identification of fungi [50,51]. ITS2 sequences are highly variable and have been adopted as the universal fungal barcode sequence [52].

The disadvantage of the amplicon sequencing approach is that the stage of PCR amplification of the sequenced region introduces an inaccuracy in the assessment of the representation of taxa in the sample under study. The existing algorithms for the selection of primers for amplification of the target DNA region cannot exclude a possible bias in the representation towards specific taxa [53]. This is easy to understand with the example of archaea. Many primers designed for bacteria, or “universal primers” are supposed to identify both bacteria and archaea present in samples. However, when the researchers used primers that were designed specifically to identify archaea, they identify a startling number of neglected species [54,55].

Untargeted shotgun metagenomic NGS analyses forego the use of specific primers or probes and provide a comprehensive characterization of complex microbial communities [56,57]. A comparison of the two approaches showed that the 16S rDNA amplicon sequencing yields quantitatively and qualitatively different results compared to shotgun metagenomics when the two techniques are used to assess the microbial community composition on the same samples. It was shown that shotgun metagenomics in terms of identified species offers a more reliable assessment [58,59,60]. Moreover, it has multiple advantages including increased detection of diversity and increased prediction of genes, as well as the opportunity to simultaneously study bacteria, archaea, viruses, and eukaryotes [59,60]. These circumstances have led to the fact that shotgun metagenomic analysis has become more and more widespread. Increasing the depth of coverage of modern sequencers to 1.8 Tbp (Illumina NextSeqX) and reducing the cost of sequencing have made deep shotgun metagenomic sequencing of rhizosphere samples available [61,62]. An additional advantage of the shotgun metagenomic analysis is that it allows reconstruction of preferential metabolic pathways, implemented, for example, in the open online service MG-RAST [63,64].

Despite the obvious advantages of shotgun metagenomics, amplicon sequencing can be useful in many cases as a cheaper and simpler method. Recently, several tools have also been developed, including PICRUSt2 [65] and Tax4Fun2 [66], to predict the functional potential of the microbiome based on 16S rRNA gene sequence data. Strictly speaking, the best choice is to use both approaches (ideally in conjunction with metatranscriptomics).

In general, bioinformatics analysis largely determines the outcome of the metagenomic functional prediction. Primary used programs have common steps, including alignment of target sequences on the reference sequences database and counting copies of functional genes. The most commonly used classifiers are BLAST, MAPSeq, QIIME, and SINTAX, while IDTAXA is new and promising [67]. Currently, fewer researchers resort to clustering sequences into operational taxonomic units (OTU), preferring to analyze exact sequence variants (ESV) or amplicon sequence variants (ASV) [68]. The choice of the reference base of nucleotide sequences also determines the microbial profile of the community. SILVA, RDP, Greengenes, and NCBI databases are used for bacterial sequence analysis [69], while UNITE and Warcup are used for fungal sequences [70]. Since online bioinformatic tools cannot solve the huge amount of data, shotgun metagenome reads are usually pre-assembled into contigs using a local computing cluster. In very rare cases, the resulting assemblies exceed the size of several gigabases, which allows them to be analyzed on the MG-RAST server. However, in addition to the assembly-based approach, metagenomic profiling without assembly is often used. In this case, the reads are mapped to preloaded databases. The most commonly used tool for taxonomic profiling of communities is MetaPhlAn [71], and the HUMAnN pipeline is used for functional annotation [72]. This approach allows for a better assessment of the functional properties of the community and does not require such large computing power as the assembly-based approach. At the same time, the disadvantage of this method is the impossibility of assigning individual metabolic pathways to a specific representative of the community. In addition, this method is poorly suited for the analysis of communities with a predominance of uncultivated forms of microorganisms.

#### 2.2.2. Application of Metagenomics to the Study of the Plant Microbiome, Breeding, and Food Security

Using metagenomic analysis, many studies have confirmed the incredibly high genetic diversity of the soil microbiome [40,41,73], including tens of thousands of bacteria, archaea [74], fungi, oomycetes, algae, protozoa, and viruses [46,75]. This diversity, on the one hand, is a challenge for plants; on the other hand, it provides significant benefits. Although many higher plants have a fairly large genome, the total information richness of their metagenome can be enormous. In addition, plant genomes are relatively conservative, and the possibility of a diverse choice of micropartners from the soil communities provides the systems with the necessary plasticity for adaptation. These communities can be regarded as the host’s extended genome, providing a huge range of potential functional capacities [76]. Cooperation with microorganisms significantly increases the potential of plants in many areas, such as nutrition, development, protection, and resistance to stress factors. After describing the various microbiomes of the rhizosphere, phyllosphere, and endophytes of many plants, it was concluded that plants possess the mechanisms of selection and proliferative support of harmless microorganisms, forming specific communities [77]. Thus, it was shown that in spatial samples in the direction of the soil–rhizosphere–rhizoplane, there is a significant decrease in the species diversity of microorganisms with an increase in their total number and biomass [78]. Communities of endophytic compartments are much less complex than those of the rhizoplane. However, although the endophytic microbiome can be altered by environmental factors (including temperature) and soil conditions such as pH and moisture [79], the plant’s immune system and features of plant biochemistry play a key role in determining the structure of its microbiome [80]. Due to this, the composition of endophytic communities in plants living under different conditions, for the most part, overlaps [81].

Although the selection mechanisms used by plants significantly enrich the plant microbiome with beneficial microorganisms, such as plant growth-promoting rhizobacteria (PGPR) [76] and disease suppressing microorganisms [76,82,83], many researchers have shown that the rhizosphere and all plant compartments often contain both phytopathogens and human pathogenic bacteria as well as producers of toxins that can enter the food chain directly from plants [84,85,86]. Moreover, if a plant disease occurs, it not only promotes the multiplication of the pathogen, but is also accompanied by dramatic changes in the entire microbiome [87]. In this regard, metagenomics is a promising tool for phytopathogen diagnosis, and food quality control, as well as developing clinical metagenomics [88].

The sequencing method currently the most attractive for metagenomics-based pathogen identification is nanopore sequencing using Oxford Nanopore Technologies (ONT) [89,90]. The advantages of this approach are that it is fast, does not require an amplification step (direct sequencing), and can be applied without prior knowledge about the pathogen since any pathogen, besides RNA viruses, can be directly detected and identified. The long reads assured by Nanopore sequencing provide additional advantages in the reconstruction of functional pathways in microbial communities and in the prediction of their species composition. The main drawback of this technology is the high error rate [91]. However, read correctness has been continuously improved, with a current modal raw read accuracy of 99.3% [92]. In addition, the combination of ONT and Illumina technologies can be used to improve the quality of sequence assembly in metagenomic studies [93]. Metagenomic sequencing with MinION^TM^ has already been used to identify fungal, bacterial, and viral pathogens on tomato and several other crops [94,95,96], and the current trend towards lower sequencing costs while improving quality suggests that direct sequencing is likely to be the next step in metagenomics [97].

As the availability of metagenomic information increases, more propositions for its capitalization becomes feasible. It was suggested that, according to the concept of personalized medicine, next-generation agriculture should aim at customizing the practices and tools tailored to the individual soil environment. Initial molecular diagnostics, including the soil microbiome report, could provide the basis for rational choice of appropriate farming treatments [98]. At the same time, since not only the microbial, but also the host side is an integral part of the interaction, the complementary response of plants to beneficial microorganisms should become an important part of future breeding programs so that next-generation plant cultivars have enhanced capacities to interact with beneficial microbes of the natural soil microbiota or of microbial inoculants [99,100]. In this part of breeding, metagenomics should afford target designation and supply information on the comparative characteristics of varieties for this trait. However, since genomic analysis does not provide information on the functional state of biological objects, a combination of genomic and transcriptomic approaches can serve as one of the key points for optimizing the plant-microbial interaction and crop improvement.

## 3. Transcriptomics 

### 3.1. Routine Transcriptome Analysis

Although most cells of an organism share the same set of genes (the genome), the transcription of these genes is specific to the developmental stage, external conditions, tissue type, etc. Transcriptomics studies the pool of RNA synthesized under certain conditions (transcriptome). The transcriptomic approach is an instrument for comparing the gene expression profiles at the level of the entire genome. The pool of mRNA (the transcripts of protein-encoding genes) is usually in the focus of most transcriptomic studies since the data on mRNA pool alteration may be mapped on the metabolic pathways, allowing the prediction of the key molecular players involved in the physiological process of interest [101].

Any transcriptomic research includes the extraction of total RNA from the samples, cDNA library preparation, and differential measuring of the transcript amounts using high-throughput methods followed by bioinformatic analysis [102]. The first attempts of the high-throughput quantitative analysis of numerous transcripts were made in the 1990s when two alternative techniques were introduced: the serial analysis of gene expression (SAGE) based on Sanger sequencing [103], and the miniaturized cDNA hybridization microarrays [104]. However, the early transcriptomic studies did not allow the profiling of the entire transcriptome because of the expensive of the procedure and lack of wholly sequenced genomes for many species, including *Arabidopsis thaliana* and *Oryza sativa*, the primary plant models. However, due to the success of genome sequencing consortiums and the emergence and development of next-generation sequencing (NGS) platforms in the mid-2000s, “true” transcriptome-wide analysis became possible. Transcriptomic analysis employing NGS techniques is considered routine.

NGS-based transcriptomics is widely implemented in various fields of plant sciences. It is used to study the processes of plant cell growth and specialization [105,106,107,108], to reveal the molecular events associated with the development of mutualistic interactions between a plant and its symbionts [109], and to study the regulation of photosynthesis [110,111] and the circadian control in plants [112], etc. NGS-based transcriptomics is often applied to plant pathology and stress studies. This approach is essential for characterizing the plant’s physiological response to pathogens and elucidating the signaling events during pathosystem development. For example, NGS revealed a candidate gene network underlying the resistance of barley to *Blumeria graminis* [113], unraveled the broad spectrum of potential molecular events associated with plant susceptible response to *Pectobacterium atrosepticum*-caused infection [114], elucidated gene expression patterns that are characteristic of compatible and incompatible interactions between potato plants and *Phytophthora infestans* [115], and pointed to the effects of viral infection on phytohormone metabolism [116], etc. In addition, NGS is also used to study non-infectious diseases and plant adaptation to unfavorable abiotic conditions. For example, the detailed transcriptome profiles of *Capsicum annuum* undergoing cold, heat, salinity, and osmotic stress were obtained [117], the high ultraviolet-caused stress response in *Arabidopsis* was characterized from the view of differential gene expression [118], changes in gene expression patterns caused by short-term flooding were revealed in orchardgrass [119], and novel pathways associated with the tolerance to hypoxia in tomato were hypothesized [120], etc.

By identifying genes highly upregulated at a particular stage of cell development, analyses of transcriptomes gave the “molecular keys” for targeted crop improvement [121,122]. The search for such “molecular keys” can be especially effective in combination with the other approaches, such as laser microdissection [123,124] or single-cell transcriptome profiling [125,126], which allow the isolation of a specific cell type at a particular stage of development. Transcriptomics combined with metabolomic/biochemical assays yields comprehensive results in studying the plant adaptation processes [127] or metabolic pathways [128,129].

Both the advantage and disadvantage of NGS transcriptomics are that a vast array of data which, on the one hand, reflect to a large extent the global picture of the target physiological process, but, on the other hand, is too extensive to be effectively “translated” into physiological language. Therefore, the interpretation of NGS transcriptomic data is complex for organisms with complex genomes, which include higher plants [130]. In addition, the regulation of a physiological process is not restricted to the expression level of an individual gene in most cases, so in the last decade, transcriptomic studies shifted their focus from single-gene level to the gene set level [131]. The necessity of considering the expression of numerous functionally related and/or cognate genes creates an additional challenge in the interpretation of NGS data.

To put the results of Differential Gene Expression DGE analysis into a physiologic context, functional gene classification is a first-choice method [131]. Gene functional classification represents a grouping of genes according to the molecular and/or physiological functions of their products. The most conventional way of functional gene classification is the use of annotations provided by curated databases keeping functional gene sets or gene ontologies along with the semantic information (i.e., relations between genes, their functions, and biologic processes) [132]. The most widely used databases are Gene Ontology (GO), Kyoto Encyclopedia of Genes and Genomes (KEGG), as well as Clusters of Orthologous Groups (COG) for prokaryotes and the euKaryotic Orthologous Group (KOG) for eukaryotes. The accession numbers of the selected database are assigned to the gene IDs by means of utilizing programming in R, Python, or SQL to map genes to functional categories. As a result, a table containing the category and gene IDs in separate columns is created. Such tables are already pre-computed for popular model plant species. For example, GO annotations of Arabidopsis can be accessed on the TAIR FTP website (ftp://ftp.arabidopsis.org/home/tair/Ontologies/Gene_Ontology/, accessed on 8 July 2021). KEGG ontology entries for the same species are available (https://www.genome.jp/dbget-bin/www_bget?gn:T00041, accessed on 8 July 2021), as well as KOG accession numbers (ftp://ftp.ncbi.nih.gov/pub/COG/KOG/, accessed on 8 July 2021). As for the non-model species, there are often no such pre-computed mappings of gene IDs to functional categories. However, there is an approach allowing the de novo gene classification by their sequences. This may be done using such tools as Blast2GO [133], BlastKOALA [134], and eggNOG [135]. When a species’ genome is mapped to the ontology/pathway database, the identifiers and names of metabolic pathways from the corresponding database are merged into one table. Some bioinformatic tools provide complete automated pipelines for gene classification. The most popular programs/web services for plant gene classification are Mercator [136], DAVID [137], PANTHER [138], and MetaCyc [139].

Automatic bioinformatics tools for functional gene classification, although very helpful, are too rough, approximate, and sometimes even inadequate to see the objective picture of the target process. Due to this, much information contained in a data array remains “hidden from the researcher’s eye”. To improve the analysis, the classification may be supplemented by using other databases (if available) and ‘manual’ targeted search of necessary information through the literature sources. In addition, the information from the specialized databases could be used for the classification of certain functional categories. For example, numerous proteins are functionally and spatially related to the plant cell wall. Two resources may be useful for finding and classifying the genes encoding such proteins: CAZy database (http://www.cazy.org/, accessed on 8 July 2021) that contains the descriptions for proteins interacting with carbohydrates (including the interactions that occur during cell wall modification), and SUBA4 database (https://suba.live/, accessed on 8 July 2021) that contains consensus annotations on the subcellular location of the Arabidopsis proteins. Transcription factors are one more abundant category of genes in plants to be classified in the transcriptomic study. There are specialized resources devoted to plant transcription factors that may aid their classification, for example, PlantTFDB (http://planttfdb.gao-lab.org/, accessed on 8 July 2021) and PlantPAN (http://plantpan.itps.ncku.edu.tw/, accessed on 8 July 2021).

Thus, NGS-based transcriptomics has become a necessary instrument for plant science because it allows us to obtain a full and comprehensive picture of gene expression under the given conditions. Large amounts of data generated in each RNA-Seq analysis are to be “translated” into physiological language. The latter is achieved through gene set functional classification by using numerous bioinformatics pipelines, general and specialized databases of gene annotations, and a “manual” assessment of the obtained results. Due to this, the progress in NGS transcriptomics largely depends on the improvement of databases for functional gene classification. The development of the improved algorithms that would contribute to a deeper interpretation of RNA-Seq data will allow us to take full advantage of the high-throughput approach to discover the molecular events occurring in plants. In addition, proper interpretation of RNA-Seq data often enables the prediction of gene products or regulatory mechanisms that determine beneficial traits of cultural plants. These traits can be exploited in the breeding process to obtain high-quality crop plant production.

### 3.2. Spatial Transcriptomics and In Situ Tissue Profiling

Today, there are 25 different approaches combining gene expression data with spatial information, which can be counted as spatial transcriptomics methods in the broad sense. Most of these spatial technologies were developed in the last decade [140]. Spatial transcriptomics methods available today are generally divided into five groups: (1) technologies based on microdissected gene expression, (2) in situ hybridization (ISH) technologies, (3) in situ sequencing (ISS) technologies, (4) in situ capturing technologies, and (5) in silico reconstruction of spatial data [140]. There is also an online resource of the classification of spatial transcriptomics methods, e.g., The Museum of Spatial Transcriptomics [141]. Despite the rapid development, most of these spatial techniques have not yet found an application in plant science. Therefore, in this review, we focus on those methods that are currently used for plants.

The first group, technologies based on microdissected gene expression, includes six methods: Laser Capture Microdissection (LCM) and its modification, named geographical position sequencing (Geo-seq); RNA-seq of individual cryosections; RNA tomography (tomo-seq); transcriptome in vivo analysis (TIVA); and NICHE-seq and ProximID [140]. One of these methods used in plant science is LCM. As soon as LCM was first adapted for plants in 2003 [142], it became a widely used method in plant science [124,143,144]. Preparation of plant material samples for LCM includes the following steps: tissue fixation, embedding, cryo- or paraffin sectioning, section mounting, laser dissection of the tissue areas, and collection of the dissected tissue fragments to obtain an amount sufficient for RNA isolation [145,146]. The most challenging part is the selection of an appropriate fixative for plant material. The best choice turned out to be Methacarn Fixation used by Shibutani et al. [147], which was then proposed for LCM by Serova et al. [148].

RNA-seq of individual cryosections involves preparation of thin cryosections, subsequent RNA isolation from individual sections, followed by sequencing and bioinformatics analysis of each sample. Such studies were carried out on tangential cryosections (15–50 μm thick) of the cambial zone of aspen (*Populus* sp.) shoots. This approach allowed the generation of transcriptome profiles of the wood-related genes in aspen with high spatial resolution. The gene expression model was named AspWood [149,150,151].

The group of ISH technologies comprises single-molecule RNA fluorescence ISH (smFISH), multiplex smFISH with sequential hybridizations (seqFISH), multiplexed error-robust FISH (MERFISH), seqFISH+, ouroboros smFISH (osmFISH), RNAscope, and DNA microscopy. A common feature of ISH technologies is that RNA molecules are not isolated from the tissue but can be visualized directly in their original environment [140]. All ISH spatial technologies are based on the development of RNA ISH, the first method that allowed the direct detection of RNA transcripts in the tissue context. The use of RNA ISH began in the early 1980s on animal tissues [152]; the technology was subsequently adapted for plant tissues [153]. The common elements of RNA ISH are as follows: tissue preparation and permeabilization; hybridization of the target RNA with nucleic acid probes; post-hybridization treatments [154]. At first, radio-labeled probes were used for hybridizations until RNA FISH was developed [155]. The first RNA FISH by Singer and Ward was based on the detection of the probe by avidin conjugated to rhodamine [155]. Horseradish peroxidase-based chromogenic (or colorimetric) RNA ISH was developed later as an alternative to RNA FISH without the use of a fluorescence microscope [154]. Both colorimetric [156] and fluorescent detection [157] of RNA by ISH became a trendy method.

Some limitations of the RNA ISH technology, such as non-linear signal amplification for RNA visualization and loss of information relating to the subcellular localization of transcripts, were overcome by some modifications: individual RNA molecules were detected by a 50-base pair fluorescently labeled DNA probe [158,159]. The RNA FISH technology involving fluorescent probes was named smFISH [159]. Despite applying this technique to the other model systems, it took about 20 years from the development of smFISH to its application to plants. The first study in plants using smFISH dates back to 2016 when smFISH was used in *Arabidopsis thaliana,* and *FLOWERING LOCUS C, COOLAIR*, and *Protein Phosphatase 2A* genes were studied in the Arabidopsis roots [160,161]. The same investigators provided a detailed protocol for smFISH on Arabidopsis roots in 2017 [162]. In another study, smFISH was compared with FISH for the detection of small RNAs [163]. The detailed protocol for smFISH for maize anthers was provided by Prof. Jeffrey Caplan in 2020 [164].

Another spatial technique called RNAscope has also been successfully applied in plant science. RNAscope was developed in 2012 as improved RNA ISH technology using branched DNA probes also called «Z-probes». Each Z-probe comprises three regions: a unique 14–25-base pair region, complementary to the RNA molecule to be detected; a short linker region and a 14-base pair tail region. When two Z-probes (double Z) carrying two different tail regions hybridized in pair to a target RNA region (50-base pair region), the two tail regions from the pair of Z-probes form a 28-base pair region complementary to another DNA molecule called the preamplifier. The preamplifier consists of 20 binding sites for the amplifier, which, in turn, contains 20 binding sites for the label probe. For example, a 1-kb length RNA molecule can be labeled by 20 Z-probe pairs and therefore by 8000 label probes. Thus, RNAscope allows a robust amplification of the signal, which in common RNA ISH can only be achieved by using antibodies binding the RNA probe [165].

The first application of the RNAscope technology with colorimetric RNA detection in plant tissues was carried out in 2014 [166]. *Phosphoenolpyruvate carboxylase* and *phosphoenolpyruvate carboxykinase* mRNAs were detected in formalin-fixed paraffin-embedded cross-sections (7 µm) of maize leaf [166]. The same colorimetric RNAscope approach was carried out in 2016 and 2018. In the first study, *Citrus tristeza* virus RNA was detected in *Citrus macrophylla* leaf petioles and roots [167]. In another study, RNA from Cassava brown streak virus was detected in stem tissues of *Nicotiana rustica* and cassava (*Manihot esculenta*) as well as in petioles and leaves of the latter species [168]. A detailed protocol for the detection of viral nucleic acids in plant tissues using the colorimetric RNAscope technique was also provided in 2020 [169]. The first RNAscope ISH using the fluorescence RNAscope technique was provided in 2020. This technology was used for single- and multiplex detections of *GAPDH* and *Rga1* mRNAs in barley leaf sections. [170].

An alternative approach to ISH technologies is promoter-driven reporter gene expression analysis using the coding sequences of enzymes (e.g., beta-glucuronidase, GUS) or fluorescent proteins cloned downstream of the target gene promoter. A glance at the plaBiPD database [171] shows that the list of sequenced plant genomes is rapidly increasing. The lists of plant species transformable by different methods [172,173] together with the broad range of cloning systems [174] are also expanding. These factors altogether make promoter-driven reporter gene expression analysis in transgenic plants easier.

In situ sequencing (ISS) includes several technologies: ISS using padlock probes; Barcode in situ targeted sequencing (BaristaSeq); Spatially resolved Transcript Amplicon Readout Mapping (STARmap), and Fluorescent in situ RNA sequencing (FISSEQ) [140]. We could not find any reports about ISS technologies currently used in plant science, so we hope that this group of spatial techniques will soon find their application to plants.

Methods including Spatial Transcriptomics (ST), Slide-seq, High-definition Spatial Transcriptomics (HDST), Nanostring GeoMx Digital Spatial Profiler, APEX-Seq, and Microfluidic Deterministic Barcoding in Tissue for spatial OMICs sequencing (DBiT-seq) are grouped into in situ capturing technologies [140]. As outlined above, all methods combining gene expression data with spatial information can be described by the term spatial transcriptomics. This term can be applied to the ST method invented 2016 in Sweden [175]. It should be noted that spatially resolved transcriptomics was selected by Nature Methods as Method of the Year 2020 [176]. As with other spatial transcriptomics methods, ST was first adapted for animal tissues. Briefly, this technique consists of the following steps. Tissue is fixed, embedded, and sectioned. Tissue sections are mounted onto glass slides printed with barcoded oligo(dT) primers, specifying the x and y coordinates of the array. Sections are stained and imaged. Tissues are permeabilized. During the permeabilization, mRNA molecules diffuse vertically down to the solid surface and hybridize with primers on the glass. Afterward, cDNA synthesis with fluorescently labeled nucleotides is performed, followed by removal of the tissue section from the glass. A cDNA footprint remains on the glass that represents the transcripts of the tissue section form and can be visualized by fluorescence microscopy. The cDNA–mRNA complexes from the area of interest can be extracted for library preparation and NGS readout [140,175]. This ST methodology was adapted for plants one year later [177]. The detailed protocol of plant tissue preparation for the analysis was also provided [178]; the authors pointed out that the protocol can be applied to any plant species with available genome or transcriptome assemblies without requiring the generation of transgenic lines.

Indubitably, ST has an advantage over the methods based on the time-consuming generation of transgenic plants (required for, e.g., fluorescence-activated cell sorting and isolation of nuclei tagged in specific cell types). A benefit of the ST over the ISS approach is the simultaneous processing of numerous plant tissue sections, thereby significantly reducing technical variability and the direct visualization of global gene expression profiles in tissue sections, options that are not provided by the LCM approach. Despite its significant strengths, ST also has three main weaknesses: it requires genomic or transcriptomic assemblies; it might be affected by the lignification types in sectioned tissues, and its resolution is currently limited to 100 μm. Thus, looking through the application of the different spatially resolved transcriptomics approaches in plant science shows that the spatial context has an unquestionable role for the deeper meaning of the biological processes in plant tissues and organs.

### 3.3. Single-Cell Transcriptomics

The transition from studying plant transcriptomes at the tissue level to the level of individual cells was an issue for plant science. To prevent this issue, methods allowing identify gene expression levels at the small cell populations were developed. These methodologies can be based on the generation of protoplasts such as fluorescence-activated cell sorting (FACS) [179,180,181] or on protoplast-free approaches such as translating ribosome affinity purification (TRAP) [182,183]; fluorescence-activated nuclei sorting (FANS) [184]; and isolation of nuclei tagged in specific cell types (INTACT) [185,186,187]. However, transcriptome analysis methods described above are applied to the entire cell population; the differences between single cells are lost.

To overcome this resolution barrier, single-cell transcriptomics was developed. A glance at the papers about using the single-cell transcriptomics in plant science showed that it became very popular (see Table S1) and is widely discussed technology today [126,188,189,190]. Several groups undertook the first attempts for single-cell transcriptome assessment in 2013 [191] and 2015–2016 [192,193]. The first single-cell RNA-seq (scRNA-seq) obtained by these studies had high technical noise. Therefore, algorithms have been developed to separate biological variation from technical noise. The creation of the first high throughput commercial droplet-based platform (10X Genomics Chromium single-cell microfluidics device) allowing generate transcriptomes from single cells [194] became the starting point for the rapid development of single-cell transcriptomics, first for animals and then for plants. The advantages of the droplet-based platform are the high yield of the harvested single cells and transgenic-free approach, which implies no time-consuming generation of transgenic plants. It should be noted that the new droplet-based approach does not replace FACS or INTACT based on generation of transgenic plants and FANS based on both transgenic and nontransgenic approaches. It increases their resolution from the small cell population to the single cell level. Successful combinations of FACS [195], FANS [196,197] and INTACT [198] with commercial droplet-based platform were reported (see Table S1). Therefore, there is no doubt that studies combining the TRAP approach with 10X Genomics barcode technology may appear soon.

All scRNA-seq approaches can be divided into two large groups: protoplast-based and protoplast-free methods. Indubitably, protoplast free based on single nuclei generation has an advantage over the methods based on protoplast generation, because protoplasting generates some forms of stress transcripts and, consequently, introduces differences in gene expression. Moreover, tissues with lignified secondary cell walls are difficult to treat with cell wall digestion enzymes. In addition, it is well known today that contacts between adjacent plant cell walls play an important role in many developmental processes, and therefore, the integrity of the cell wall is essential.

Both protoplast-based and protoplast-free scRNA-seq methodologies have the following steps. Generation and collection of protoplasts or nuclei followed by validation assay are the first and the second general steps. A different way could make protoplasts or nuclei validation before sc/snRNA-seq. For example, protoplasts can be observed under a light microscope and if necessary, any excess of debris or unprotoplasted tissues removed with an additional washing step. As a further step, a protoplast suspension can be filtered through a 40-μm cell strainer to eliminate any debris and clumped cells. The same approaches can be used for single nuclei validation: additional washing steps or DAPI-stained nuclei sorting using FANS are applied to remove any cell debris or organelles. Counting of the protoplasts can be done by a hemocytometer or by dilution to a small numbers of cells (100–150 cells). The viability of the protoplasts can be analyzed by DAPI staining, and unstained protoplasts are used for further analysis. Alternatively, in order to detect dead and damaged cells, the bioinformatics approach can be applied using the percentage of mitochondrial, chloroplast, and ribosomal genes [199].

Then, scRNA isolation and amplification are performed. The fourth step is scRNA library construction and sequencing. Eight platforms for both scRNA isolation and library preparation are available today [199,200]. Chromium System (10x Genomics, Pleasanton, CA, USA); Nadia Instrument (Dolomite Bio, Royston, Hertfordshire, UK); InDrop System (1CellBio, Watertown, Massachusetts, USA); ddSEQ Single-Cell Isolator (Illumina Bio-Rad single-cell sequencing solution, Illumina, San Diego, CA, USA; Bio-Rad Laboratories, Hercules, CA, USA); Tapestri Platform (Mission Bio, South San Francisco, CA, USA) and BD Rhapsody Single-Cell Analysis System (Becton Dickinson, Franklin Lakes, NJ, USA) belong to high throughput platforms. ICELL8 Single-Cell System (Takara Bio, Kusatsu, Shiga, Japan), C1 System, and Polaris (Fluidigm Corporation, South San Francisco, CA, USA) are medium-throughput systems [200]. Platforms can also be divided into two main groups. The first is a droplet-based approach using commercially available instruments to sort cells/nuclei to the individual droplets (e.g., Chromium; Nadia; InDrop; ddSEQ, and Tapestri). The second group is the well-based approach combining FACS or FANS into 96-well plates followed by cDNA library preparation (e.g., Smart-seq2 protocol). The comparative analysis of all scRNA-seq platforms available today is in appropriate references [199,200].

The main problem of the droplet-based platforms is the cell/nuclei size effect. Briefly, several protoplasts or nuclei can fall into one drop. For protoplasts from reporter plant lines (e.g., Arabidopsis), the well-based approach can be performed to overcome this issue. DAPI staining can reveal nuclei from wild-type plants, and after that, removing of doublets can be easily done with well-based method. Doublets can also be detected bioinformatically at the data processing stage. Several packages are available today for this aim such as DoubletFinder [201], Scrublet [202], and DoubletDecon [203,204]. DoubletFinder and Scrublet were successfully applied to plant scRNA-seq data (see references 5, 11, 15, 27, 32, and 37 in Table S1).

Thereafter, it started with scRNA-seq read processing and cell filtering including read trimming, alignment, and unique molecular identifier (UMI) counting steps to generate a UMI count matrix. The last stage includes sequential steps such as dimensionality reduction, cell-type classification, and differential expression analysis followed by trajectory inference, pseudotime analysis, and batch-effect correction. All of the above steps require extensive bioinformatics training. Bioinformatics software for data processing and the stages in which it is applied is reviewed in the appropriate references [188,199]. There is also the website for 897 scRNA-seq bioinformatics tools [205]. It should be mentioned that an avalanche-like growing amount of plant scRNA-seq data was a new challenge for plant science; therefore, a plant scRNA-seq database was created [206]. Summarizing the single-cell data available today, it could be said that the first step toward the Plant Cell Atlas creation was done in the past three years.

The data obtaining by the spatial and single-cell transcriptomics approaches increase in an overflowing manner. Therefore, the data generated by these approaches indubitably can be used for the ever-growing needs of humanity. In terms of food and nutritional security, transcriptomics data are invaluable for expression analysis of economically important genes and improvement of environmental conditions used in breeding programs.

### 3.4. Metatranscriptomics

DNA analysis of plant–microbial communities has significant limitations, which, first of all, are determined by the difficulties in identifying living and metabolically active organisms. In this regard, metagenomics, reflecting the potential of a community, must be supplemented with methods that express its real activity. First of all, DNA approaches can be supplemented with RNA metabarcoding [207] and metatranscriptomics (RNA-seq) [208]. This approach solves the problem of extracellular DNA, dead and dormant cells, and also better reflects the activity of processes occurring in plant-associated microbiomes. With the application of RNASeq, it is now possible to not only measure known transcript targets but also to discover previously unknown transcripts and resolve viral–host relationships, including RNA viruses. Further, metatranscriptomics provides some additional tools. For example, the use of polyA tail hybridization during sample preparation allows targeting of only eukaryotic mRNAs [209,210].

At the same time, metatranscriptomics has only recently entered the cohort of methods of omics technologies used to study plants. This is partly due to methodological challenges arising from the complexity of microbial communities, the large dynamic range of transcript expression, and the short half-life of RNA [55]. The complex microbiomes are frequently characterized using metagenomic sequencing, but only a few studies have performed metatranscriptomics to decipher active microbes from plant-associated communities. However, these works may require a rethinking of existing approaches. In one of them, metatranscriptomes showed that *Verrucomicrobia*, which is found in high abundance in soils and rhizosphere, is not always as active as its numbers might suggest. The authors showed that the high abundance of *Verrucomicrobia* is partly due to the presence of metabolically inactive organisms [211]. Another study used a comparative metatranscriptomic approach to assess the taxonomic and functional characteristics of the rhizosphere microbiome of wheat plants grown on soils that were suppressive and non-suppressive against the plant pathogen *Rhizoctonia solani* AG8. The taxonomic composition of the active rhizosphere community based on mRNA sequences was very similar between the metatranscriptome libraries of the suppressive and non-suppressive samples. Differential expression analysis showed that 65 bacterial species and one archaeal strain differed significantly in their gene expression between suppressive and non-suppressive soils [212].

Metataxonomy is of particular interest for the study of plant-microbial pathosystems since the development of a disease is often not determined by the presence of a pathogen but is conditioned by the dynamics of processes of plant–microbial and microbial–microbial interactions affecting all members of the community. Thus, in the study of wilting of olive trees (*Olea europaea*) by metatranscriptomics, it was shown that the disease, although caused by a phytopathogenic fungus *Verticillium dahliae*, is driven not by a single species, but by a polymicrobial consortium that also includes natural endophytes of the olive tree. This community contains both biotrophic and necrotrophic organisms that alternate and live together during the infection. The progression of infection initiates a dynamic cascade of events that culminates in altered gene expression patterns in all interacting organisms. In addition, opportunistic organisms appear to profit not from plant tissues, but from new emerging populations of microorganisms [213].

Another advantage of metatranscriptomics is that it provides more relevant information about gene regulation than pure culture studies. Clearly, the environment in a plant host differs substantially from in vitro models, which may affect gene expression. In recent work by Jo et al. (2020, [214]), DNA shotgun, sequencing, and RNA-sequencing with poly(A) selection were used to study the microbial community from mummified peach fruits. It was found that many bacteria and fungi live together in the affected tissues in the presence of several dominant species. Moreover, most of them exhibit active transcription of many genes. The authors note that RNA sequencing provides better detailed information for microbial communities; however, combining DNA and RNA sequencing results increased the diversity of microorganisms including fungi, bacteria, viruses, and viroids.

In the cited works, most attention was paid to the interaction of plants and phytopathogenic fungi. Despite the great interest in transcriptome studies of endophytes and pathogenic bacteria inside the host, such works remain rare, for which there are objective reasons for the study of bacterial infections in animals and humans [215]. First of all, these studies may be limited due to bacterial mRNA not being polyA tailed and this in turn results in low yields of expressed mRNAs when they are extracted. Second, a large genome size difference is translated into different amounts of RNA. Although each infected host cell usually contains several bacteria, in RNA preparations obtained from infected tissues, the proportion of bacterial RNA in most cases does not exceed 5% [215]. Another challenge is the high complexity of RNA libraries with a high content of ribosomal and transport RNA and the lack of reliable tools for enriching libraries without losing information and material [192]. In this regard, obtaining sufficient coverage of bacterial transcriptome sequences against a eukaryotic background becomes a limiting parameter of the experiment. To solve this problem, two approaches selective for the 5′ end of primary transcripts, for the differential enrichment of mixed RNA preparations with prokaryotic transcripts, were developed [216,217]. However, these methods have not yet received widespread use due to the technical complexity and difficulty in interpreting the results. Meanwhile, the increasing performance of modern NGS equipment makes it costly but feasible to achieve a reliable result without library enrichment. Recently, total transcriptome profiling was performed on tobacco plants infected with *Pectobacterium atrosepticum*. Deep-coverage RNA sequencing made it possible to compare bacterial traits under in planta and in vitro conditions and to reveal potential players that participate in switching from the stealth to brute force strategy of the pathogen [218]. A similar strategy was used to assess potential mutualisms between microorganisms and the seagrasses *Zostera marina* and *Zostera japonica* [219]. Further, in-depth RNAseq has been used to study the diversity and function of microorganisms in relation to carbohydrate metabolism of ripe watermelon fruits [220]. Undoubtedly, as the technique of high-throughput sequencing improves and its cost decreases, the number of such works will dramatically increase.

In conclusion of this chapter, we can say that the task of transcriptomics for breeding programs is the functionalization of genomic information. In other words, transcriptome resources should provide an understanding of what information in the genome is most important for development, high yields, stress response, and disease resistance, ultimately assisting in the development of improved crop varieties by breeding. However, addressing this issue requires a wider shift from laboratory experiments to field studies providing information on systemic transcriptome patterns of crop productivity that will be compatible with genomic selection (GS) and other approaches that are already broadly used in breeding.

## 4. GWAS, Genomic, and Phenomic Prediction

Instead of examining phenotypic observations for a small number of variables, the biological systems can be studied based on a global analytical approach. To study complex biological processes holistically, it is imperative to take an integrative approach that combines multi-omics data to understand better a situation, system, or process [221,222]. This data integration could be defined as using several sources of information, which can compile several information levels, such as the genome, transcriptome, proteome, metabolome, fluxome, and phenomics.

An approach that combines different molecular signs is needed, which enables prediction, that is essential to understand how the variations in phenotypic traits are explained at a genomic level between plants [223,224]. Several sources of information can be used, such as high-performance omics technologies: DNA arrays, microarrays, protein chips, and mass spectrometry (LC-MS and GC-MS), among others [225,226,227]. Plant genomics studies and breeding programs have benefited from advances in high-throughput OMICs technologies, which has allowed us to investigate the function of thousands of genes and genomic regions [222,228].

The number of breeding programs that have already implemented genomic-assisted breeding has increased considerably in recent years [229,230,231]. The use of this approach has been facilitated by the development of high-throughput genotyping techniques (such as genotyping by sequencing and DNA chip arrays) in various important crop species, including maize [232,233,234], tomato [235,236], wheat [237,238], and rice [239,240], among others. Identification of genomic regions associated with a complex trait is based on a model in which many loci underlie the trait of interest continuously and in which non-genetic factors may also be important [241,242]. In this sense, a wide range of analytical methods is now available, which can be used in various stages of a breeding program, which aim to optimize the components of the selection gain equation; i.e., increase selection intensity, accuracy, and genetic advance, and reduce the time for recycling new strains, hybrids or cultivars.

With the development of high-throughput genotyping techniques, the use of the genome-wide prediction (or genomic selection: GS) approach to increase breeding progress by shortening generation intervals has been proposed, in which a large number of molecular markers is employed. Their effects are estimated on a training set (TS) of phenotyped and genotyped individuals [243]. GS is a method proposed by Meuwissen et al. [244] to increase dairy cattle improvement programs efficiency. GS was developed as an alternative method to pedigree-based BLUP (Best Linear Unbiased Prediction), incorporating genomic data. Unlike the classic Marker-Assisted Selection (MAS), in GS, the effects of thousands of markers are predicted simultaneously, even though these are not individually significant for a trait of interest. According to Daetwyler et al. [245], GS can increase genetic gain ranges since individual genetic merits are estimated with greater precision. Even though GS does not allow us to identify the function of the possible genes controlling a quantitative trait, the predictive models provide a short-term selection criterion for those individuals who have a better performance. Furthermore, GS has improved the understanding of the genetic architecture of phenotypic traits and even implements ecological restoration plans [246]. The most widely known GS methods are based on the Bayesian framework: Bayes A, Bayes B, Bayes Cπ, Bayesian LASSO (Least Absolute Shrinkage and Selection Operator), Bayesian Ridge Regression (BRR) [244,247,248], and methods based on the classical BLUP: Genomic-BLUP (GBLUP) [249]) and Ridge Regression BLUP (RR-BLUP) [244]. Regarding the assumptions of analysis, RR-BLUP and GBLUP assume that the markers have the same variance and each marker contributes a small effect to the prediction model (infinitesimal model). The prediction via GBLUP is performed similarly to BLUP, with the difference that in the BLUP method, the pedigree matrix is replaced by a kinship matrix constructed from molecular markers. On the other hand, RR-BLUP is a multiple regression method in which the markers are thousands of regressors that explain the variation of a phenotypic trait. In contrast, the methods Bayes A and Bayes B assume that each marker has a variance, and loci explain the phenotypic variance with effects of different magnitude [129].

The Bayesian methods differ from the a priori distributions that are established and the degree of adjustment that is used [229,250]. Briefly, in the Bayes A method, the marginal distribution of marker effects is a scaled-t density, in which this density is implemented as an infinite mixture of scaled-normal densities, due to computational convenience [208]. The variance of each marker (*v_mi_*, *i*=1, …, *n* markers) is considered to be distributed as a scaled inverse Chi-square distribution. Bayes B uses a mixed distribution with a mass of zero, such that the prior distribution of the effects of all markers (*m_i_|v_mi_,* *π*) is given by 0, with probability *π*, and ~*N (0, v_mi_)* with probability 1–*π*. In this case, the prior of *v_mi_*, is equal for all markers, which corresponds to a scaled inverse Chi-square distribution. In the Bayes Cπ method, all markers are considered to have a common variance and promote the selection of variables similar to the Bayes B method. The effects of the markers (*m_i_|v_m_,* *π*) for Bayes Cπ, are assumed as: ~*N (0, v_m_)* with probability 1–*π* = 0. On the other hand, the Bayesian LASSO method considers that the effects of the molecular markers (*m_i_*) are distributed *a priori* according to a double exponential (*DE*): *p (m_i_|λ, v_e_) = DE (m_i_|0, λ, v_e_)*, where λ corresponds to a regularization parameter, and *v_e_* corresponds to the residual variance. BRR considers that model regressors (molecular markers) have a common variance (*v_m_*), so that those regressors with the same allelic frequency explain the same proportion of the additive variance and have the same contraction effect [208]. In this case, the effect of markers (*m_i_*) is distributed as: *m_i_|v_m_~N(0, v_m_)* in which the common variance (*v_m_*) is assumed to be a scaled inverse Chi-square distribution. In any genomic selection study, it is recommended to test various available methods [251], and these must be countered in terms of their precision or predictive abilities. However, if the researcher approximates how many loci could explain a trait variation, he/she could use a particular method. For example, the Bayes B model bases its analytical assumptions on highly heritable traits whose variation is explained by large-effect loci [129]. Bayes A represents an option for traits that are controlled by a moderate number of genes. Some studies have shown that Bayesian methods tend to be more accurate than GBLUP when the training and validation populations are genetically weakly related [252,253].

On the other hand, high-throughput phenotyping (HTP) techniques have been developing enormously over the last two decades. Both approaches, genomics and phenomics, have promised to revolutionize the field of plant breeding [254,255,256,257]. An integrated approach that combines high-density genotyping and HTP can improve the prediction accuracy of quantitative traits in plants. For instance, Mackay et al. [258] proposed a strategy for genomic prediction. The accuracy of assessment in the reference population for a primary trait is increased by incorporating data from high-throughput field phenotyping platforms. Assume the traits collected from HTP platforms are genetically correlated with the primary trait. In that case, such traits could be considered secondary traits to improve rates of genetic gain for the primary trait in genomic selection. Using secondary traits collected from HTP platforms would also help predict primary traits at early growth stages, as they could be phenotyped ahead of the primary trait [259].

**Figure 1:**
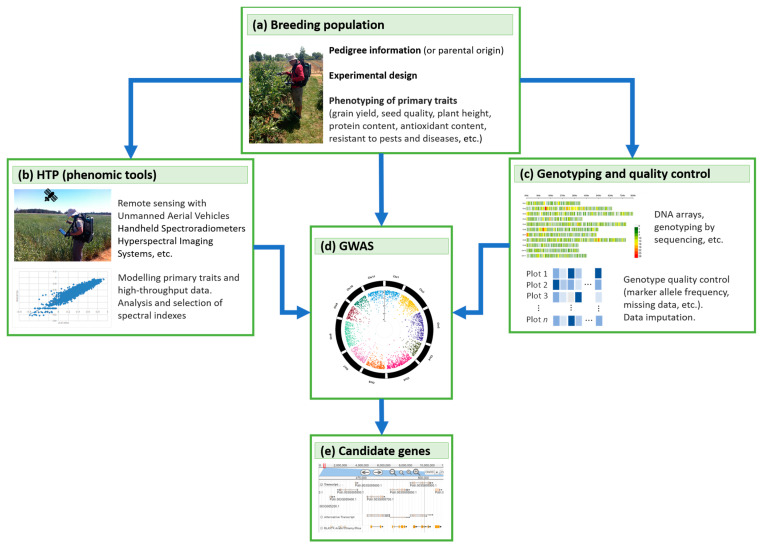
Overview of an integrated approach using genome-wide association study (GWAS) and spectral information for dissection of complex traits in a crop species (adapted from Feng et al. [261]). (**a**) A traditional breeding population (e.g., association panel, progeny trials, inbreed lines, etc.) is evaluated based on primary traits (traits of interest such as grain yield, seed quality, plant height, protein content, antioxidant content, resistance to pests and diseases, etc.). Pedigree information, experimental design, and other information can be considered. (**b**) One or more high-throughput phenotyping (HTP) platforms can be used to improve gene discovery, such as remote sensing with Unmanned Aerial Vehicles (UAVs), handheld spectroradiometers, hyperspectral imaging systems, etc. Hyperspectral or spectral indices are modeled with the primary traits selected according to their predictive ability. (**c**) Plants are genotyped using genotyping platforms; genotype quality control is performed. (**d**) GWAS of hyperspectral and spectral indices and traditional traits is performed. SNP or haplotype data can be used for a better dissection of a complex trait. (**e**) Candidate gene identification is carried out based on GWAS results. This combined strategy could provide additional gene discovery capabilities, as Feng et al. (2017) reported [261].

High-throughput genotyping technology and phenotyping platforms have enabled large-scale marker-trait association analysis, such as GWAS, to precisely dissect the genetic architecture of plant traits [260]. In plant species, few studies have evaluated the combined use of HTP techniques and GWAS. In earlier work, Feng et al. [261] showed that an integrated data approach using hyperspectral imaging and the GWAS platform could provide spectral and genetic insights into the natural variation in rice. In that study they used a high-throughput hyperspectral imaging system (HHIS), which was developed to obtain hundreds of hyperspectral indices at a whole-plant level during tillering, heading, and ripening stages; these indices were then used to quantify traditional agronomic traits and to explore genetic variation. An illustration of a basic scheme of the approach carried out by Feng et al. [261] is shown in Figure 1, i.e., GWAS combined with high-throughput phenotyping platforms. According to their results, the authors concluded that this combined strategy could provide additional gene discovery capabilities of complex traits.

Alternatively, Rincent et al. [262] proposed the concept of “phenomic selection” by using near-infrared spectroscopy (NIRS), a high-throughput phenotyping technique, to indirectly capture endophenotypic variants for predicting breeding values of complex traits. They evaluated the efficiency of NIRS to make predictions of complex traits in wheat and poplar, using these traits instead of molecular markers (i.e., computing spectra data as random effects). In an integrative data study, Krause et al. [263] proposed a multi-kernel GBLUP approach to genomic selection that uses genomic marker-, pedigree-, and hyperspectral reflectance-derived relationship matrices to model the genetic main effects and genotype × environment (G  ×  E) interactions across environments within a bread wheat breeding program. This study demonstrated the potential of using hyperspectral imaging to predict a primary trait (grain yield, in this case) within a multi-environment context and support further studies on integrating hyperspectral reflectance phenotyping into breeding programs. Additionally, the advantages of the integration of high dimensional data, such as hyperspectral reflectance, into relationship matrices for use in GBLUP, have been addressed by Krause et al. [263], among which is the possibility of integrating different types of highly dimensional phenotypes for prediction, such as ionomics and metabolomics data. This opens the possibility of obtaining more holistic molecular perspectives from crops compared to traditional approaches.

Crop genetic improvement programs have benefited from advances in high-throughput technologies, which have allowed us to investigate the regulation and function of thousands of genes and genomic regions involved in adaptation to environmental challenges, including climate change. In fact, with current genomic and phenomic techniques, it has been possible to recover substantial portions of plant genetic diversity, which is a key input for genetic improvement programs, food security, and conservation programs. In this sense, genome-wide studies along with phenomic techniques have revolutionized the field of crop breeding, in plant species key for food security.

## 5. Plant Epigenetics and Epigenomics: OMICs Studies (Methylome by WGBS and Histone Modifications by ChiP-Seq)

Genetic composition of every species is the result of hundreds of years of evolution. Genomes of the organisms are very stable, and fluctuations in genetics, known as mutations, are rare and cannot provide an adequate response to the changes in the environment. In contrast, the epigenetic regulation is much more dynamic and allows immediate response to the environmental fluctuations; heritability and reversibility allow epigenetics to be a first “go to” mechanism of response to environment. Epigenetic regulation consists of covalent modifications of DNA and histones, affecting the transcriptional activity of various genes without changing the DNA sequence [264]. In addition, differential expression of non-coding RNAs that regulate gene expression at transcription and posttranscriptional levels is also considered epigenetic in nature. Finally, nucleosome repositioning or eviction, histone variants, and rearrangements of chromosomal (chromatin) domains are also part of epigenetic regulation.

The dynamic nature of chromatin and the activity of non-coding RNAs allow for alterations in cellular activities controlled through epigenetic changes in gene expression, affecting biological processes such as seed germination, flowering, embryo formation, and responses to biotic and abiotic stresses [265]. Besides immediate response to stress, epigenetic modifications also allow acclimation and adaptation to environmental stresses and can lead to inheritance of such modifications, resulting in epimutations. DNA and histone methylation are major mechanisms of epigenetic regulation [266]. The DNA methylation occurs mainly at cytosine bases in three sequence contexts CG and CHG (symmetric), and CHH (asymmetric), where H represents A, T, or C [267]. In plants, de novo methylation in all cytosine sequence contexts is established by an RNA-directed DNA methylation (RdDM) pathway via DRM2 (DOMAINS REARRANGED METHYLTRANSFERASE 2), a homologue of DNMT3, and thereafter is maintained by various methyltransferases including MET1 (METHYLTRANSFERASE 1; CG context), a homolog of DNMT1; CMT3 (CHROMOMETHYLASE 3; mainly CHG context), a plant specific methyltransferase; and DRM2 and CMT2 (CHROMOMETHYLASE 2; mainly CHH context) [268,269]. DRM2 is accountable for the maintenance of CHH methylation in short euchromatic regions, short Transposable Elements (TEs), and the edges of long TEs, while CMT2 is accountable for the maintenance of CHH methylation in pericentromeric heterochromatin and the bodies of long TEs [270]. Methylation in the CHG context via CMT3 often requires methylation of histone H3 at lysine 9 by H3K9 methyltransferases such as SUVH4/ KYP, SUVH5, and SUVH6 [271]. In the RdDM pathway, Pol IV (homologs of RNA polymerase II) synthesizes the long single-stranded RNA molecules (ssRNA). Then, ssRNA are converted into double-stranded RNA (dsRNA) by RDR2 (RNA-DEPENDENT RNA POLYMERASE 2) and further processed by DCL3 (DICER-LIKE 3) nucleases into small 24 nucleotide short interfering RNAs (siRNAs). These siRNAs are then loaded onto the AGO4 (ARGONAUTE 4) complex and interact with Pol V synthesized nascent transcripts from target loci, which results in DRM2 recruitment and DNA methylation at the target loci [267]. In addition to methyltransferases, the chromatin remodeling factor DDM1 (DECREASE IN DNA METHYLATION 1) also plays a crucial role in maintaining cytosine methylation in CG and non-CG contexts. Mutation in the DDM1 resulted in a 70% reduction in the global cytosine methylation level [272], predominantly in heterochromatic H3K9me2-enriched regions [269]. Mutations in genes involved in DNA and histone methylation showed reductions in global cytosine methylation consequently resulting in more active chromatin [269,273]. DDM1 and RdDM pathways together with RdDM-independent pathways (such as CMT3-SUVH4/KYP) are accountable for methylation and silencing of nearly all transposable elements (TEs) and genome stability [273,274,275]. DNA demethylation is mediated via a subfamily of 5-methylcytosine glycosylases comprising REPRESSOR OF SILENCING 1 (ROS1), DEMETER (DME), DEMETER-LIKE2 (DML2), and DML3 [276].

Epigenetic regulation is also performed through histone modifications. Histone modification mainly occurs in histone tails via covalent post-translational modification of various amino acids, including mono/di/trimethylation, phosphorylation, acetylation, and ubiquitylation. These histone modifications cause either decondensed (open) or condensed (closed) chromatin conformations, which activate or repress transcription of genes, respectively [277]. Histone methylation is one of the best-studied histone modifications in plants [278]. In Arabidopsis, histone methylation mainly occurs at Lys4 (K4), Lys9 (K9), Lys27 (K27), Lys36 (K36), and Arg17 (R17) of histone H3 and Arg3 (R3) of histone H4 [279]. These methylation types have different effects on chromatin configuration. H3K4me and H3K36me are permissive chromatin marks generating decondensed (open) transcriptionally active chromatin configuration [279], whereas H3K9me and H3K27me are repressive marks creating a condensed (closed) transcriptionally inactive chromatin configuration [280,281]. H3K9me2 functions as a silencing mark linked to DNA methylation [282], while H3K27me3 represses the expression of many genes targeted by Polycomb repressive complex PRCs [283]. Histone demethylation is mediated by various histone demethylases including Jumonji proteins [284].

This part of the review discusses the effect of chromatin modifications (DNA and histone methylation) mainly on transcriptome regulation, leading to phenotypic resilience and stress adaptation in plants. Here, we will discuss the studies that comprise the OMICs of analysis (global DNA methylome analysis by WGBS and genome-wide histone methylation by ChIP-Seq analysis).

### 5.1. Regulation of Plant Stress Response by Dynamic Changes in DNA Methylation: Analysis of Global DNA Methylome by WGBS

Global methylome analysis can be examined by whole genome bisulphite sequencing (WGBS). Changes in DNA methylation include differentially methylated positions (DMPs), where single cytosines are involved, and differentially methylated regions (DMRs), where multiple cytosines in a given area are considered [285]. Numerous studies have reported global changes in DNA methylation in response to abiotic and biotic stresses and identified the stress-responsive genes regulated by DNA methylation [286]. Wang et al. [287] provided insight into the DNA methylation dynamics in carbon reserve remobilization of rice stems, demonstrating that soil drying increases this remobilization, and suggested an association between DNA methylation and gene expression in rice stems during grain filling. They generated whole-genome single-base resolution maps of the DNA methylome in the stem and observed an increased in global DNA methylation during grain filling under soil drying. Further, they reported that a hypermethylated/up-regulated transcription factor MYBS2 inhibited MYB30 transcription and possibly enhanced β-Amylase5 expression, promoting subsequent starch degradation in rice stems under soil drying conditions. A hypermethylated/down-regulated transcription factor of ERF24 was predicted to interact with and thus decrease the expression of abscisic acid 8’-hydroxylase 1, consequently increasing abscisic acid concentration under soil drying. Al-Harrasi et al. [288] studied the genome-wide differential methylation in response to salinity stress exposure in the date palm root epigenome. They identified salinity responsive genes that are regulated by differential methylation. Overall, the global DNA methylation increased in response to salinity, and hypermethylated-DMRs were observed explicitly at the CHG and CHH contexts, and a positive correlation was observed between CHG/ CHH methylation status and gene expression. Similarly, Yaish et al. [289] reported methylome analysis of *Medicago truncatula* root tissue exposed to saline stress and observed that the average global methylation level was increased in all sequence contexts in response to salinity. They observed 77% of DMPs in the CHH context, while only 9.1% and 13.9% in the CHG and CG contexts, respectively. Gene expression analysis did not reveal a consistent relationship between the level of CG methylation and the transcription abundance of some salinity responsive genes, indicating the involvement of other epigenetic regulation. Heat stress induced a global disruption of DNA methylation in cotton anthers, mainly causing CHH hypomethylation in heat-sensitive cotton varieties [290]. The decrease in genome-wide DNA methylation might result in the interruption of glucose- and ROS- producing metabolic pathways, which may lead to microspore sterility [290]. Similarly, heat stress induced global DNA hypomethylation, especially in the CHH context in soybean root hairs tissue [291]. *Brassica napus* cultured microspores displayed hypomethylation in the CG and CHG contexts under heat-shock treatment [292]. In contrast, the heat-sensitive genotype of rapeseed exhibited significantly higher global genome methylation levels than the heat-tolerant genotype under heat stress [293].

A H_2_O_2_ overproducing transgenic tobacco line with biotic and abiotic stresses resistance was studied to determine the genome-wide DNA methylation changes [294]. The WGBS analysis revealed a total of 9432 DMPs predominantly in the CHG context with a trend toward hypomethylation. Out of 9432, 1117 sites were associated with genes, and 83 genes were differentially expressed in the transgenic tobacco and were associated with respiration, energy, and calcium signaling pathways [294]. Another study reported global methylation changes in the genome of commercial apple (*Malus x domestica*) under water deficit in drought-sensitive and drought-tolerant cultivars. The interplay between the altered expression profile of water-deficit responsive genes and methylation changes was noted [295]. Rajkumar et al. [296] studied the methylome of various rice cultivars (desiccation-tolerant: Nagina 22, salinity tolerant: Pokkali and sensitive: IR64) and observed that methylation in the CHH context was most dynamic under desiccation and/or salinity stress conditions in these rice cultivars. Further, they found that hypomethylation in the CHH context was correlated with higher gene expression under desiccation stress in Nagina 22. In contrast, hypermethylation in the CHH context was associated with higher gene expression under salinity stress in Pokkali. The results also showed that the stress-responsive genes harbored DMPs (epimutations) between the sensitive and tolerant rice cultivar(s), suggesting the role of epialleles in abiotic stress responses.

Recently, Li et al. [297] performed mulberry WGBS under drought stress and found that the global methylation levels under drought stress was higher than those in the control. They identified 3243 genes with differential methylation and expression patterns that were enriched in biological processes such as catalytic activity, cellular process, metabolic process, and response to stress stimulus. Further, Qian et al. [298] reported the genome-wide DNA methylation pattern of maize leaves in response to heat exposure and identified candidate genes that were enriched in spliceosome, homologous recombination, RNA transport, ubiquitin-mediated proteolysis, and carbon metabolism pathways. At the same time, Sun et al. [299] investigated changes in DNA methylation under salt stress by using the Methylated DNA Immunoprecipitation Sequencing (MeDIP-seq) method in maize. They discovered that the methylation in the CG context was lower than that in the CHG and CHH contexts. A total of 4402 differentially methylated regions (DMRs) between stress samples and the control, in which hypomethylation was predominant under the salt stress, were observed. These DMR-associated genes were enriched in biological processes such as cellular processes, metabolic processes, and signal transduction. An et al. [300] explored the DNA methylation changes during seed maturation through whole-genome bisulfite sequencing. CHH methylation levels in cotyledons changed greatly from 6% at the early stage to 11% at the late stage, and the majority of the DMR-associated genes in the CHH context were transcriptionally downregulated as seeds matured; these genes were preferentially associated with DNA replication and cell division. Atighi et al. [301] showed a significant global hypomethylation as a crucial plant defense mechanism in response to nematode or bacterial pathogen infection in rice and tomato. The authors also demonstrated that DNA hypomethylation in the CHH context was associated with a reduced susceptibility to root-parasitic nematodes in rice.

Environmental stresses have an impact on the directly exposed parental plants as well as on their progenies via parental effects and/or transgenerational effects, a phenomenon also known as stress priming [302]. Several reports demonstrated the ability to maintain the memory of stress exposure throughout ontogenesis and transmit this memory to the offspring [303,304,305,306,307]. Ou et al. [308] reported transgenerational inheritance of modified DNA methylation patterns in three successive generations of rice with an enhanced tolerance to heavy metal stress. Zheng et al. [307] showed that multigenerational drought improved the drought adaptability of offspring in upland fields. By using WGBS analysis, they discovered drought-induced non-random epimutations and further demonstrated the maintenance of epimutations in advanced generations. The genes related to transgenerational epimutations directly participated in stress-responsive pathways.

### 5.2. Regulation of Plant Stress Response by Dynamic Histone Modifications: Analysis of Genome-Wide Histone Modifications by ChiP-Seq 

Genome-wide histone modifications are examined by chromatin immunoprecipitation (ChIP) with sequencing (ChIP-Seq). ChIP-seq is mainly used to determine the active (such as H3K4me, H3K36me, and H3K9ac) and repressive (such as H3K9me and H3K27me) chromatin marks genome-wide and analyze their impact on transcriptome regulation; these data can then be correlated with data on phenotypic stress resilience and adaptations. Alterations of histone modifications in response to stress have been well-documented [302,309]. Van Dijk et al. [310] studied the genome-wide dynamic changes in active histone mark mono/di/tri H3K4 methylation in Arabidopsis in response to dehydration stress, revealing that permissive marks H3K4me were predominantly located on gene bodies. In response to dehydration, the H3K4 tri methylation level changed more robustly than mono- and di-methylation levels in stress-responsive genes. Yan et al. [311] provided the genome-wide profiles of three active histone marks (H3K4me3, H3K36me3, and H3K9ac) in *Paulownia fortunei* (dragon tree) under phytoplasma stress by using ChIP-Seq. They found that H3K4me3, H3K36me3, and H3K9ac were mainly enriched in the genic regions and revealed 1738, 986, and 2577 genes associated with these marks, respectively, in response to phytoplasma infection; most of these genes were involved in metabolic pathways, biosynthesis of secondary metabolites, phenylpropanoid biosynthesis, plant-pathogen interaction, and plant hormone signal transduction. They further showed that differential histone methylation and acetylation only affected a small subset of phytoplasma-responsive genes. Further, Yan et al. [312] also studied these histone active marks under recovery from phytoplasma infection after methyl methane sulfonate treatment. They detected 365, 2244, and 752 genes associated with disease recovery enriched in H3K4me3, H3K36me3, and H3K9ac marks, respectively. These genes displayed higher expression and were involved in calcium ion signal transduction, abscisic acid signal transduction, and ethylene biosynthesis. Sun et al. [313] studied the dynamics of genome-wide transcription and histone methylation patterns in soybean roots under salt stress and identified 8798 genes with differential H3K27me3 histone repressive marks and found that the downregulation of genes under salt stress was strongly associated with the de novo establishment of H3K27me3.

The biological relevance of histone modification in secondary xylem development in *Eucalyptus grandis* using ChIP-seq revealed that H3K4me3 and H3K27me3 putative bivalent domains are enriched in the late lignification pathways processes of xylogenesis but not in the early secondary cell wall polysaccharide deposition process [313]. Zeng et al. [314] reported that cold stress elevated chromatin accessibility via establishment of bivalent H3K4me3-H3K27me3 histone marks in gene body regions of active genes, which may enable recruitment of the regulatory proteins required for gene regulation in cold stress. The differential gene expression was associated with cold stress-induced Dnase I hypersensitive sites enriched in genic regions [314]. In *Magnaporthe oryzae* (a fungal plant pathogen), dynamic changes at H3K27 with respect to methylation (repressive mark) and acetylation (active mark) play a crucial role in the regulation of genes that are vital for host infection responses [315]. Histone demethylase SlJMJ6 enhanced fruit ripening of tomato by eliminating methylation of H3K27 in fruit ripening-related genes (such as RIN, ACS4, ACO1, PL, TBG4, DML2, etc.), which are mainly involved in transcription regulation, hormone signaling, ethylene biosynthesis, and cell wall degradation [316].

### 5.3. Application of Epigenetics and Epigenomics to Improve Crop Germplasm

Now it is well established that some epigenetic variations are stable and can faithfully transfer to the offspring. Differential epigenetic marks appear as a potential resource of variations in agronomic traits such as plant fitness, flowering time, seed dormancy, stress resistance, and yield. The heritable nature of epigenetics variations also suggests a crucial role in plant domestication and evolution, consequently emerging as a potential tool to improve crop germplasm [317,318]. The epigenetic variability can be induced by chemical treatments (5-AzaC, zebularine, trichostatin A, etc.), mutations in epigenetic pathway-related genes, by abiotic and biotic stresses, and targeted epigenetic modification by gene editing [319]. These natural and induced epigenetic variations can be screened to decipher their association with important agronomic traits for epigenetic-assisted breeding and gene editing programs to develop superior next-generation crops.

#### 5.3.1. Epigenetic-Assisted Molecular Breeding

The stability and heritability of epialleles and epigenetic markers epi-QTLs (DMPs and DMRs) across generations and their association with agronomic traits (epi-traits) led to epigenetic-assisted crop breeding via artificial selection. Several approaches have been utilised to develop epi-populations with epigenetic variations to establish epigenetic-assisted breeding program of crops, including the use of mutant and RNAi-suppressor lines [320,321], recurrent or recursive epi-selection [322,323], hybrid mimics [324], epigenomic selection [325,326], stress priming [302], and epigenome editing [302].

Epigenetic recombinant inbred lines (epiRILs) of Arabidopsis were derived from crossing of the wild type with a homozygous mutant deficient in DNA methyltransferase 1 (*met1*) or chromatin remodeler decrease in DNA methylation (*ddm1*), which showed segregation and heritability of modified DNA methylation patterns together with associated phenotypic diversity [327,328,329,330,331]. The analysis of epigenetic variations (DMPs and DMRs) in epiRILs revealed that epigenetic variations can act as epigenetic quantitative trait loci (epi-QTLs) and can participate in development of superior crop variety with important agronomic traits via natural and artificial selection [330]. The epigenetic variation in segregating populations resulted in variations in complex traits such as flowering time, root length, and yield [330]. The epiRILs displayed altered biomass production under biotic stress, which was partly driven by complementarity among epigenotypes [332]. The recursive selection on epigenetic features of energy use efficiency exhibited higher yield potential and inheritance of acquired methylation patterns and agronomic traits in canola [322]. These studies demonstrated that epigenetic variations are amenable to natural and artificial selection and could be exploited effectively in breeding program to develop superior crop germplasm.

The MSH1 system is an important approach to exploit epigenetic variations in epigenetic-assisted breeding to develop superior crops. Plant MutS HOMOLOG1 (MSH1, a homolog of the bacterial DNA repair gene MutS) encodes a dual-targeted protein that suppresses illegitimate DNA recombination and localizes in the mitochondria and plastid [333]. The depletion of MSH1 influences both mitochondrial and plastid properties [334]. The T-DNA insertional mutation in the *MSH1* gene in Arabidopsis exhibited altered plant growth associated with changes in plastid properties. The *msh1* mutant altered phenotype includes leaf variegation, reduced growth rate, delayed flowering, extended juvenility, different floral morphology, aerial rosettes, changed perennial growth behavior, as well as tolerance to abiotic stresses [334,335,336,337]. The altered expression of MSH1 can lead to developmental reprogramming associated with genome-wide changes in DNA methylation, specifically, elevated non-CG hypermethylation of pericentromeric genomic intervals that increases phenotypic plasticity in response to environmental changes [338,339]. Further, Shao et al. [340] demonstrated extensive changes in transcriptome, specifically changes in the expression of genes related to defence response, abiotic stress, MAPK cascade, circadian rhythm, and phytohormone pathways in msh1 mutants.

The complex developmental reprogramming phenotypes have been produced in both monocot and dicot crops by using RNAi-suppression of MSH1 [320,336,341]. The altered phenotype is subsequently inherited independently of RNAi transgene segregation, involving epigenetic modifications independent of genetic changes [336,341]. Crossing of the modified plant, either the *Arabidopsis thaliana* msh1 mutant or the sorghum RNAi-suppression lines of MSH1, with respective isogenic wild-type produced heritable enhanced vigor phenotypes in subsequent lineages [339,341]. The vigor phenotypes displayed rapid growth, early flowering, increased biomass, and improved seed yield. Yang et al. [320] used the MSH system to develop epigenetic-assisted breeding program for tomatoes. They developed the MSH1 RNAi-suppressed populations of tomato ‘Rutgers’ with wide-ranging epigenetic variations that were heritable in subsequent lineages independent of the MSH1-RNAi transgene. The crosses of the modified lines to respective isogenic wild-type produced vigor phenotypes including better growth, earlier ripening, higher yields, and heat tolerance under both greenhouse and field conditions. Further, they showed that the vigor phenotypes were graft transmissible and were partially obviated by application of exogenous methylase inhibitor (5-azacytidine), which confirmed the epigenetic nature of the vigor phenotypes.

Recently, Raju et al. [321] established the MSH1 system for epigenetic-assisted soybean breeding programs to achieve higher yield. They developed MSH1 epi-populations by crosses between wild-type and msh1-acquired soybean memory lines, with a wide-ranging variation in multiple yield-related traits in both greenhouse and field trials. The epi-F_2:4_ and epi-F_2:5_ lines showed an increase in seed yield compared to the wild-type. However, the epi-F_2:6_ line showed a yield trait similar to that of the wild-type, which suggests that novel epigenetic variation can be inherited for at least three generations. Furthermore, the authors showed a reduced epitype–environment interaction, indicating higher yield stability and a lesser effect of environmental constraints. Transcriptome analysis of epi-lines identified genes involved in numerous metabolic pathways responsible for vigor yield trait across generations. Overall, the authors presented the potential of the MSH1-system in epigenetic-assisted breeding for improved yield traits in soybean.

In addition to the MSH1 system, it is also viable to identify natural epi-populations (epialleles) that impact plant growth. Hauben et al. [322] produced breeding lines with enhanced energy use efficiency (EUE) for higher seed from individual plants of an isogenic canola population, and their self-fertilized progenies were recursively selected for respiration intensity. These generated populations were genetically identical, but different in the epigenetic context. Furthermore, both the modified epigenetic patterns and the specific agronomic traits of the selected lines were heritable. A 5% yield increase on top of heterosis was observed in hybrids derived from parent lines selected for high EUE. Overall, the authors demonstrated that artificial selection at the plant level with recursive manner permitted the enhanced yield potential associated with epi-trait in canola. The tomato fruit-ripening variant colorless nonripening (cnr) appears to be a naturally occurring epiallele of CNR, which encodes an SBP-box transcription factor. In the variant, CNR expression is silenced by promoter hypermethylation to inhibit normal fruit ripening [342]. These natural epigenetic variations impacting plant growth may be important to develop important agronomic traits in crops.

Recent advances in the epigenomics resources (global methylome and ChIP-Seq data) of major crops have enabled high throughput screening of genome-wide epigenetic marks associated with important agronomic traits that could be utilized in epigenetic-assisted breeding with full potential.

#### 5.3.2. Precise Epigenome Editing Approach

The genome-wide identification of epigenetics marks associated important agronomic traits facilitated the development of superior crops through precise and targeted epigenetic modifications by using the latest epigenome editing tools. There are several advanced engineered DNA-binding domain-based epigenome editing tools available such as zinc fingers (ZFs), transcription activator-like effectors (TALEs), and the endonuclease-deficient Cas9 (dCas9) protein that can be used in combination with either activator or repressor functional domains to introduce permissive or repressive epigenetic marks at targeted loci [343]. The deactivated version Cas9 (dCas9) is used in epigenome editing because the endonuclease activity of the CRISPR-Cas9 system is not required. The epigenome editing tools consist mainly of two domains, a targeting DNA-binding domain and a functional domain, such as methyltransferase or demethylase. The targeting domain can be constructed on ZF proteins, TALEs proteins or the clustered regularly interspaced short palindromic repeats (CRISPR-dCas9) system. The functional domain can contain epigenetic modifier proteins such as DNA or histone methyltransferase, DNA or histone demethylase, histone acetyltransferase, or histone deacetylase to produce specific epigenetic marks at a targeted locus. More details about these tools can be found in Section 7.

In 2014, Johnson et al. [344] used ZFs accompanied with SUVH2, a protein essential to the RdDM pathway for an unmethylated epiallele *fwa-4* of *FWA* in Arabidopsis, to induce locus specific DNA methylation. The outcome indicated that Pol V is recruited through the methyl-DNA binding SUVH2, leading to methylation in the targeted locus, which resulted in gene silencing and early flowering phenotype in the edited plants. Later, in 2018, Gallego-Bartolomé et al. [345] designed a fusion construct of the catalytic domain of the human demethylase TET1cd (TEN-ELEVEN TRANSLOCATION1cd) and zinc finger (ZF) to target the *FWA* promoter, which demonstrated highly efficient targeted demethylation, *FWA* up-regulation, and a heritable late-flowering phenotype. The authors also designed the fusion construct ZF–TET1cd to target methylated regions of the *CACTA1* transposon, which showed targeted demethylation and changes in expression.

Recent advances of the application of CRISPR-Cas9 technology also facilitated induction of site-specific DNA methylation. Vojta et al. [346] developed a CRISPR-Cas9-based tool for site-specific DNA methylation consisting dCas9 and a functional domain of the DNA methyltransferase DNMT3A and demonstrated targeted CG methylation in a ∼35-bp wide region. Further, the authors showed that multiple guide RNAs could target multiple adjacent sites, which enabled methylation of a larger region of the promoter of the target loci IL6ST and BACH2, which resulted in decreased expression. The DNA methylation establishment was specific for the targeted region and heritable across mitotic divisions. The CRISPR-dCas9 system can be used for gene silencing when fused with the DNA methyltransferase protein [347,348] and gene activation when fused with demethylase [349,350]. Gallego-Bartolomé et al. [345] developed a CRISPR/dCas9-based targeted demethylation system using the TET1cd and a modified SunTag system. Similar to the ZF–TET1cd fusions, the SunTag–TET1cd system is able to target demethylation and activate gene expression when directed to the *FWA* or *CACTA1* loci. Papikian et al. [351] used the dCas9-SunTag system for the site-specific manipulation of DNA methylation in *Arabidopsis*. The authors present a CRISPR-based methylation targeting system for plants with the catalytic domain of the *Nicotiana tabacum* DRM methyltransferase, which efficiently targets DNA methylation to specific loci, including the *FWA* promoter, triggering a developmental phenotype, and the *SUPERMAN* promoter. Recently, Nuñez et al. [352] developed CRISPRoff, a programmable epigenetic memory tool consisting of a Cas9 fusion protein that establishes DNA and histone methylation. The authors took advantage of the fact that the establishment of epigenetic marks does not require induced double-strand breaks. Fusion of functional domains of DNA-modulating enzymes with catalytically inactive Cas proteins (dCas) can activate or inactivate gene expression in mammalian cells [353,354]. The authors showed that N-terminal fusion of functional domains of de novo Dnmt 3A (Dnmt3A) [355] in conjunction with the catalytically inactive cofactor Dnmt3L works more effectively with dCas9 to add a methyl group to cytosine on CpG dinucleotides. The silencing cascade is initiated by the simultaneous C-terminal fusion of the Krüppel-associated box (KRAB) domain. Thus, CRISPRoff exhibits highly specific DNA methylation, and gene silencing is maintained through cell division and is thus inherited by the next generation. CRISPRoff’s ability to set heritable epigenetic marks associated with gene repression enables multiple applications, including genome-wide screens, multiplexed cell epi-engineering, enhancer modification, and deciphering the epigenetic mechanism of inheritance. In addition, because the system is reversible, CRISPRon enables the reactivation of epigenetically controlled genes.

The stability and heritability of epigenetic variations (epimutations) across generations and their association with agronomic traits (epi-traits) led the path to epigenetic-assisted crop breeding and epigenome editing to improve crop germplasm for food security. The availability of the epigenomic data of major crops enabled high throughput screening of genome-wide epigenetic marks associated with important agronomic traits that could be utilized in epi-breeding/epigenome editing programs with full potential to develop superior crops to achieve food security for everyone.

## 6. Gene Editing Techniques for Food and Nutritional Security

To feed an increasing population under more complex and less predictable climatic and environmental conditions, traditional breeding methods are not sufficient to develop new varieties quickly and accurately. The biotechnological harnessing of targeted gene modification using molecular scissors has opened up new possibilities and expanded the breeder’s toolbox. The technology can be used better to adapt forage and food crops to climatic changes. It can protect regional varieties more quickly from new pathogens that were not previously relevant in the growing region. Unique traits can be introduced into existing breeding material much more swiftly and precisely, without considering the “linkage drag” that often occurs in classical mutation breeding. Targeted silencing or modification of gene expression can generate a crop from a wild ancestor (de novo domestication; for review see Fernie and Yan, 2019 [356]), e.g., tomato [357]. Landraces can be converted into cultivars [358,359]. All this leads to improved food and fodder crops and more security in the population’s supply.

Increasingly comprehensive information, such as knowledge of genetic information, proteins, and secondary metabolites, allows targeted modification of one or more genes. Increasing phenotyping leads to digital data linking quantitatively measurable traits to the genetic information, identifying candidates for, e.g., resource utilization, etc. Improved models provide possible candidates that improve the appearance, aroma, or shelf life of foods through targeted modification. Plants can enrich certain beneficial ingredients such as the oleic acid content (canola, [360]) or anthocyanins (tomato, [361]) to increase nutritional value and human or animal health. Allergens or substances that limit human or animal tolerance can be eliminated (celiac disease, phytate). This list could be extended indefinitely; however, this is outside the scope of this review article.

Several platforms for targeted gene modification have been established in the last decade. Many originate from bacterial systems, but it has been recognized that broad application becomes possible by utilizing individual elements. The three best-known platforms are zinc finger nucleases (ZFNs), transcription activator-like effector nucleases (TALENs), and clustered regularly interspaced short palindromic repeats (CRISPR)-associated 9 (Cas9) endonucleases. ZFN and TALENs utilize the non-specific DNA-cleaving activity of the FokI catalytic domain. The site-specificity is achieved by coupling DNA-binding domains to the FokI domain. In ZFN, these are polypeptides, each of which binds to a DNA triplet through a loop structure [362]. The loop shape is formed by zinc as a bridging element, which is how these proteins got their name. Although there are many such zinc finger peptides, the complex production limits the application of the technology. TAL effectors were discovered in *Xanthomonas* bacteria [363], and it was recognized that these effectors have a conserved sequence of 33 to 34 amino acids that allows specific binding to host DNA. Only two positions (Repeat Variable Diresidue) are variable and responsible for the specificity of binding to the four nucleotides of the DNA. However, the TALEN modules’ production is more complex since, for each target nucleotide, one peptide has to be attached to the other.

The CRISPR/Cas system based on the bacterial immune system also consists of two components [364]. In contrast to the polypeptide-based binding to DNA, RNA molecules are responsible for the specificity. Similar to FokI, the Cas protein also cuts relatively non-specific double-stranded DNA using its HNH and RuvC-like cleavage domains. The RNA molecules used in biotechnology are a fusion of single guide RNA (sgRNA) and tracr RNA (trRNA) and are called guide RNA (gRNA). While sgRNA recognizes the target region, trRNA is needed for binding to the Cas protein.

All platforms have in common that they target a user-selected region in the target genome, and then a double-strand break is induced. Since various environmental factors can also cause double-strand breaks, eukaryotic cells have developed different repair mechanisms to repair them efficiently and without errors. However, since a double-strand break is induced again after successful repair, a defective repair occurs after a not yet precisely determined number of cycles, which on the one hand impairs gene function, but also in most cases, prevents the binding of the molecular scissors. Molecular biologists take advantage of this circumstance either to understand the role of a gene by specific knockout or to use this knowledge to modify crop plants in a targeted manner. Breeders can profit from such a method as it allows the modification of a given crop genome by copying naturally occurring or induced mutations but avoiding the simultaneous integration of unfavorable traits via cross-breeding or the undetected random mutations after chemical or radiation mutagenesis. The platforms could also integrate foreign DNA in a preselected genomic context, clearly generating a genetically modified organism (GMO). The outcome is classified into three categories (SDN-1 to 3, SDN—site-directed nuclease). SDN-1 refers to random mismatch repair following DSB induction and NHEJ-mediated repair. SDN-2 uses molecular scissors and provides a repair template from the same host, differing in one or a few nucleotides. Both are indistinguishable from nature and should be classified as non-GMO. SDN-3 includes all other events that introduce foreign DNA or shuffle the host DNA in a way that does not occur in nature. Therefore, only those events should be treated as GMOs.

The selection of the target region can influence specificity. Gene families often have conserved regions but also have sequence regions that differ within a family. This fact can be used in both directions. One chooses a unique region if one wants to knock out or alter only one gene of a gene family. If, on the other hand, an entire gene family is to be switched off, such as the α-Gliadins of wheat, a region is selected that is conserved in as many or all gene copies as possible [365]. The platforms also differ on this point. In TALENs, the binding domain is coupled to the non-specific FokI domain, which is only active as a dimer. Thus, two modules are needed, which increases the target region. Since a stretch of 40–50 nucleotides is thought to be rare or even unique in a given genome, specificity increases and thus, the off-target risk is reduced. This modular design makes it universal and allows targeting of any genomic sequence, which is limited in CRISPR/Cas technology by the presence of a protospacer-associated motif (PAM, NGG for SpCas9 [366]) directly adjacent to the target sequence.

To overcome this drawback, Cas proteins from other organisms (Cas12a recognizing 5′-TTTTN [367], *Staphylococcus aureus* Cas9 (SaCas9) recognizing 5′-NNGRRT [368], *S. thermophilus* Cas9 (StCas9) recognizing 5′-NNAGAAW [366] or modified Cas9 proteins evolved (e.g., xCas [353], SpCas9-NG [369]). In contrast to TALENs, CRISPR/Cas technology often combines two short oligos for cloning, which is much simpler and can be performed in almost any laboratory. The partially increased off-target activity is quickly resolved in plants by selecting the transgene-free progeny with the desired modifications. If undesirable phenotypic changes occur, such a plant can be easily eliminated.

The result of the induced double-strand break is predominantly insertions or deletions (Indels) caused by the most commonly used repair mechanism, non-homologous end joining [370]. These lead to alteration of a protein’s reading frame and thus often to premature arrest, preventing or severely impairing protein function. Deletions of 3, 6, or 9 nucleotides allow the targeted alteration of an amino acid sequence (functional domain), allowing the study of altered or attenuated protein function. This effect is similar to RNAi technology (for review, see Lindbo, 2012 [371]), but this is genetically fixed and does not require crossing a threshold necessary in RNAi, making the results more reproducible. Simultaneous transfer of a synthetic repair template can provide another avenue for targeted gene modification. Homology-dependent repair uses this synthetic fragment and integrates it at the site marked by the double-strand break. This allows targeted allelic replacement of preferred gene variants. However, this approach has the disadvantage that only a tiny proportion (about 5%) of the mutant cells, receive the desired change. Exemplarily, this was shown in barley by altering the emission spectrum of a fluorescent reporter gene (GFP-YFP) [372]. In corn, conferring tolerance to herbicide was used to establish the method [373]. 

Targeting multiple genomic regions allows simultaneous manipulation of different traits. There have been many developments and improvements in this regard (see review Najera et al., 2019 [374]). The impressive number of 107/109 genes encoding caffeic acid O-methyltransferases could be edited using a single TALEN pair in sugarcane (*Saccharum officinarum*) [375]. More recently, the modification of eight genes in *N. benthamiana* and 12 genes in Arabidopsis using CRISPR/Cas technology was reported [376].

For some prominent targets, several of the previously mentioned technologies have been successfully applied. For example, agronomically significant powdery mildew resistance in wheat has been achieved by targeted knockout of the MLO locus using TALEN and CRISPR/Cas [377]. Other examples include reducing the phytic acid content in corn [378] and the enrichment of anthocyanin in tomatoes [361]. A review of other product traits improved using genome editing methods can be found here [379].

Targeted gene editing methods have been continuously expanded in recent years. Base editors [380] and prime editing [381] allow more precise and predictable editing of a target sequence. One of the latest developments in the field of targeted genome modification is chromosome engineering. Here, targeted induction of double-strand breaks can lead to inversion of specific chromosome segments. For example, in the model plant Arabidopsis, it was shown that a 1.1 Mb fragment on chromosome 4 could be inverted by the targeted use of SaCas9 [382]. This makes regions accessible for the process known as a crossover, allowing new variability through crossbreeding.

In summary, gene editing provides breeders access to a new resource of genetic diversity. In addition, this approach offers two key advantages: speed and precision. The most significant progress using technologies has been made in rice, wheat, and corn. Here, there are many improvements in product quality and tolerance to biotic and abiotic stresses (for a review, see Kumlehn et al., 2018 [383]; Schindele et al., 2020 [384]; Ganie et al., 2021 [385]). There is a need to catch up due to the lack of genome sequence information and efficient transformation technologies in vegetables and fruits. There are positive examples such as tomato (summarized in Ku and Ha, 2020 [379]), and virus-resistant cucumber [386], modified mushrooms [387], and apples [388] have also been reported. However, in general, there is still much potential for development. A much bigger problem, however, is the different evaluations of gene-edited crops. In some parts of the world, plants that do not contain additional DNA elements and are thus indistinguishable from classical mutation breeding are not overly regulated. Europe has imposed high hurdles with a general classification as a genetically modified organism (GMO), thus practically preventing their cultivation [389]. In times of global feed and food flows, this creates unnecessary uncertainty, as it is also impossible to guarantee or trace how such plants were made. This European classification indirectly influences 3rd-world countries using these technologies, leading to further uncertainty in food production.

## 7. Conclusions

The preeminent advances in OMICs technologies over the past two decades, resulting from the advancement of molecular biology, have revolutionized biological science, adapting it to global digitalization [390]. The advent of big data available for machine processing has led to an explosive growth in bioinformatics as a promising new field. At the present stage, new analytical tools are used to move from the accumulation of information to its use for constructing new genomes and phenotype prediction [24,391,392]. The results of this shift remain to be seen, but the feasibility of such scenarios has already been pledged by the explosive development of advanced genome editing tools [393]. The next step will likely be the optimization of supraorganism level systems and communities of organisms, the adaptive potential of which has not yet been properly assessed by us; however, it is this potential that can provide not only a further increase in productivity but also a decrease in the need for the use of chemicals [394,395].

In this work, it was impossible to provide an exhaustive overview of all the achievements of the OMICs technologies, implemented to replenish the arsenal of breeding. Moreover, their integration into practical plant breeding programs deserves a separate consideration. At the same time, it should be emphasized that only in integration with scientific discoveries in many areas of crop production and if additional efforts are made to fill the knowledge gaps [396] can new opportunities in phenomics, genomics, and bioinformatics make effective use of genetic resources of agricultural crops and the improvement of breeding strategies more feasible.

## Data Availability

All relevant data are within the paper and its Supplementary Materials files.

## Supplementary Materials

The following are available online at https://www.mdpi.com/article/10.3390/plants10071423/s1, Table S1. List of single cell transcriptomics approaches in plant science.
