# Supplementary material for: OMICs, Epigenetics, and Genome Editing Techniques for Food and Nutritional Security
Source: Plants (Basel). 2021 Jul 12;10(7):1423. doi: 10.3390/plants10071423 (PMC8309286; doi:10.3390/plants10071423)
Supplement: Supplementary file 1 [file plants-10-01423-s001.zip › plants-1239217-supplementary.pdf]

Table S1. List of single cell transcriptomics approaches in plant science

Species	Organ	Sequencing method	Platform for single cell sorting	Platforms for cDNA libraries preparation and sequencing	Number of finally analyzed cells/nuclei	Bioinformatics soft and pipelines	Publication year	Reference
**Arabidopsis**	*pWOX5∷GFP* and *pGL2∷GFP* transgenic lines roots	scRNA-seq	manually sorting of the single GFP positive cells into PCR tubes	Illumina platform and reagents	not available	GSNAP; htseq-count; «affy» package; TopGO	2013	[1]
	pWOX5∷GFP and wt root tips cutted at a distance of 130 µm above the columella	scRNA-seq	manually single cell sorting using glass mouth pipette	Illumina platform and reagents	24 cells from quiescent centre; 7 cells from stele	bowtie; NGSUTILS; CEL-Seq; Index of Cell Identity (ICI) algorithm	2015	[2]
	root meristems of the *pAHP6::CFP* and *pWOL::CFP* transgenic lines: uncut meristems and at 3 h, 16 h, 46 h after the cut	scRNA-seq	FACS into 96-well plates (FACS Aria or Sony SY3200)	Illumina platform and reagents	238	bowtie; NGSUTILS; MAS5; getAllSpec and getIdentity functions; ICI algorithm	2016	[3]
	1 cm wt root tips (6 d old)	scRNA-seq	10x Genomics Chromium device	10x Genomics Single Cell 3′ Reagent Kit v2; Illumina platform	4727	Trimmomatic; STAR; HTSeq; DEseq2; Cell Ranger; Seurat; Monocle; SCODE; Cytoscape; NetworkAnalyzer	2019	[4]
	whole wt roots (7 or 8 d old)	scRNA-seq	Chromium Single Cell Gene Expression Solution (10x Genomics); 10x scRNA-Seq platform		3121	Cell Ranger; Monocle; Scrublet; Velocyto		[5]

Species	Organ	Sequencing method	Platform for single cell sorting	Platforms for cDNA libraries preparation and sequencing	Number of finally analyzed cells/nuclei	Bioinformatics soft and pipelines	Publication year	Reference
Arabidopsis	wt root tips (5 d old)	scRNA-seq	10x Genomics Chromium device	10x Genomics Single Cell 3′ Reagent Kit v2; Illumina platform	7522	Cell Ranger; Seurat; ComBat; Monocle	2019	[6]
	whole wt roots (5 and 7 d old)	scRNA-seq	Drop-seq platform	Illumina platform and reagents	12198	Drop-seq_tools v1.12; Seurat; Monocle; ICI algorithm; STAR Aligner; DEseq2		[7]
	whole roots with *VND7* inducible expression	scRNA-seq	Drop-seq platform	Illumina platform and reagents	250	Drop-seq_tools v1.12; Seurat; ICI algorithm; STAR Aligner		[8]
	0.5 cm wt root tips (10 d old)	scRNA-seq	10x Genomics Chromium device	10x Genomics Single Cell 3′ Reagent Kit v2; Illumina platform	7695	Cell Ranger; Seurat; Monocle		[9]
	computational analysis of available Arabidopsis root single cell datasets						2020	[10]
	wt and IAA14 low degradation plants: primary root regions with lateral root primordia induced by root bending: 8 h and 12 h post-bending	scRNA-seq	Chromium Single Cell Gene Expression Solution (10x Genomics); 10x scRNA-Seq platform		6658	Cell Ranger; Monocle; PhenoGraph; Leidan community detection algorithm; Scrublet; PANTHER		[11]
	cotyledons (5 d old)	scRNA-seq	10x Genomics Chromium device	10x Genomics Single Cell 3′ Reagent Kit v3; Illumina platform	13999	Cell Ranger; STAR Aligner; Monocle; Seurat; Cytoscape		[12]

Species	Organ	Sequencing method	Platform for single cell sorting	Platforms for cDNA libraries preparation and sequencing	Number of finally analyzed cells/nuclei	Bioinformatics soft and pipelines	Publication year	Reference
**Arabidopsis**	whole aerial tissue or first true leaves from 10 d old reporter lines	scRNA-seq	10x Genomics Chromium device	10x Genomics Single Cell 3′ Reagent Kit v2 and v3; Illumina platform	18000	clustree package; Seurat; scVelo; Slingshot	2020	[13]
			FACS into 96-well plates (FACS Aria II)	Smart-seq2 protocol for cDNA libraries; Illumina platform	500			
	seed endosperm at 2, 3, 4 or 5 days after pollination	snRNA-seq	FANS into 96-well PCR plate	Smart-seq2 protocol for cDNA libraries; Illumina platform	1437	Trim Galore v.0.4.1; STAR Aligner; bamCoverage; SC3; TopGO		[14]
	0.5 cm wt, *shortroot-2*, *scarecrow-4* root tips (5 d old)	scRNA-seq	10x Genomics Chromium device	10x Genomics Single Cell 3′ Reagent Kit v3; Illumina platform	96000	Cell Ranger; kallisto; bustools; BUSpaRse; BSgenome; ScrubletFinder; COPILOT; Seurat; Trimmomatic; STAR Aligner; FastQC; DEseq2; FastQN; CytoTRACE; scVelo; ICI algorithm		[15]

Species	Organ	Sequencing method	Platform for single cell sorting	Platforms for cDNA libraries preparation and sequencing	Number of finally analyzed cells/nuclei	Bioinformatics soft and pipelines	Publication year	Reference
Arabidopsis	diploid and isogenic tetraploids expressed cell-specific *promoter:nGFP* in ovules	scRNA-seq	manually single cell collection using micromanipulator, microcapillary pipette and inverted microscope	Smart-seq2 protocol for cDNA libraries; Illumina platform	not available	NGS QC Toolkit; STAR Aligner; SciPy; *log2* and *prcomp* functions in R	2020	[16]
	computational analysis of available Arabidopsis root single cell dataset in order to study genes regulated transition from mitotic cell cycle to endocycle							[17]
	root tips of 6 d old seedlings: wt, mutants and transgenic reporter lines	scRNA-seq	FACS into 96-well plates (FACS Aria II)	10x Genomics Single Cell 3′ Reagent Kit v2; Illumina platform	5145	Cell Ranger; STAR Aligner; scran, scater and Dyno R packages; Seurat		[18]
	whole root of the INTACT line *UBQ10:NTF::ACT2:BirA*	snATAC-seq	10x Genomics Chromium Single Cell ATAC Solution platform; Illumina platform		5283	Cell Ranger; Seurat; Louvain clustering		[19]
	whole wt root	sc/snRNA-seq, snATAC-seq	FANS into 10× Genomics microfluidic chip (FACS Aria II)	10x Genomics Single Cell 3′ Reagent Kit v3; Illumina platform	10548	Cell Ranger and Cell Ranger ATAC; Seurat; HiSat2; PicardTools MarkDuplicates; bedtools	2021	[20]

Species	Organ	Sequencing method	Platform for single cell sorting	Platforms for cDNA libraries preparation and sequencing	Number of finally analyzed cells/nuclei	Bioinformatics soft and pipelines	Publication year	Reference
Arabidopsis	*bri*^{TRIPLE} and *bri*$^{T\text{-}RESCUE}$ primary seedling roots (6 d old)	scRNA-seq	FACS into 96-well plates (FACS Aria II)	10x Genomics Single Cell 3′ Reagent Kit v3; Illumina platform	10574 (*bri*^{TRIPLE}); 12335 (*bri*$^{T\text{-}RESCUE}$)	Cell Ranger; STAR Aligner; scater; Seurat	2021	[21]
	embryos of the *pWOX2::NLS-GFP*, transgenic line	snRNA-seq	FANS into 96-well plates (FACS Aria III)	Smart-seq2 protocol for cDNA libraries; Illumina platform	534	cutadapt; kallisto; Monocle; PANTHER; UMAP		[22]
	mature wt leaves (6 w old)	scRNA-seq	10x Genomics Chromium device	10x Genomics Single Cell 3′ Reagent Kit v2; Illumina platform	5230	Cell Ranger; Seurat		[23]
	5 mm wt root tip (10 d old); endosperm from 5 DAP ovules	snRNA-seq	10x Genomics Chromium device	10x Genomics Single Cell 3′ Reagent Kit v3; Illumina platform and MinION flow cell	1186	Cell Ranger; Scanpy; Guppy; minimap2; Sicelore; Blastn		[24]
	⅓ of the root (transgenic reporter lines)	scRNA-seq	FACS into 96-well plates	Smart-seq2 protocol for cDNA libraries; Illumina platform	19 (phloem cells only)	HiSat2; SAMTools; Cuffquant; Cuffnorm; Monocle; Scanpy; Louvain clustering		[25]
	whole seedling (10 d old)	snRNA-seq	FACS using ICELL8: MultiSample NanoDispenser	SMARTer ICELL 3′ chemistry for cDNA libraries; Illumina platform	3348	bcl2fastq; ICELL8 mappa analysis pipeline; Seurat; RSEM; GENIE 3; Monocle		[26]

Species	Organ	Sequencing method	Platform for single cell sorting	Platforms for cDNA libraries preparation and sequencing	Number of finally analyzed cells/nuclei	Bioinformatics soft and pipelines	Publication year	Reference
**Arabidopsis**	wt seedlings: shoot apices (7 d and 14 d old) and 3rd leaf (18 d old)	scRNA-seq	10x Genomics Chromium device	10x Genomics Single Cell 3′ Reagent Kit v3; Illumina platform	36643	Cell Ranger; DoubletFinder; Seurat; harmony; Palantir; Scanpy; Monocle; Velocyto.R; Velocyto CLI; AgriGO; SCODE; FastQC; MultiQC; Fastp; DEseq2	2021	[27]
**maize**	germinal cells, covering a week of development from the day after archesporial cell specification to early zygotene of meiotic prophase I	scRNA-seq	manually sorting into 8-tube PCR strips	Illumina platform and reagents	not available	HiSat2; SC3; RaceID3; SNN-cliq; Seurat; SINCERA; AgriGO	2019	[28]
	mix samples (3-4 seedlings; 3 tassel or ear primordia; 12-14 root tips; 12-14 crown root samples; ~30 axillary buds)	snATAC-seq	10x Genomics Chromium device	10x Genomics Single Cell 3′ Reagent Kit v2; Illumina platform	not available	Cell Ranger ATAC; AGPv4; samtools view; MarkDuplicates; smooth.spline; Zoo; Fastp; Ciciero; DEseq2; LASSO; fgsea	2020	[29]

Species	Organ	Sequencing method	Platform for single cell sorting	Platforms for cDNA libraries preparation and sequencing	Number of finally analyzed cells/nuclei	Bioinformatics soft and pipelines	Publication year	Reference
**maize**	2 w old seedlings: ~3-mm of the SAM including the two or the six most recently initiated leaf primordia (SAM + P2; SAM + P6)	scRNA-seq	Cel-Seq2 protocol (for SAM + P2); 10x Genomics Chromium device (for SAM + P6)	Cel-Seq2 protocol (for SAM + P2); 10x Genomics Single Cell 3′ Reagent Kit v3 (for SAM + P6); Illumina platform (for both sample sets)	327 (SAM + P2); 12967 (SAM + P6)	celseq2 pipeline; Seurat; AgriGO; Monocle	2020	[30]
	distal portion of leaf 2 (from 1 to 7 cm, as measured from the tip) from V2 stage plants	scRNA-seq	10x Genomics Chromium device	10x Genomics Single Cell 3′ Reagent Kit v3; Illumina platform	3763/3242	Cell Ranger; STAR Aligner; Seurat	2021	[31]
	5-10 mm developing ears	scRNA-seq	10x Genomics Chromium device	10x Genomics Single Cell 3′ Reagent Kit v2; Illumina platform	12525	Trimmomatic; STAR Aligner; DoubletFinder; EmptyDrops; scran; rcvd, ccd and UMAP packages; MetaNeighbour; EGAD		[32]

Species	Organ	Sequencing method	Platform for single cell sorting	Platforms for cDNA libraries preparation and sequencing	Number of finally analyzed cells/nuclei	Bioinformatics soft and pipelines	Publication year	Reference
**Physcomitrella**	leaf	scRNA-seq	manually single cell collection using micromanipulator, microcapillary pipette and inverted microscope	Thermo Fisher and New England Biolabs chemistry; Illumina platform	not available	cutadapt; UMI_SC; Trimmomatic; bowtie 1.12; RSEM; SinQC; Cytoscape; BinGO; Monocle; Hmisc	2019	[33]
**Populus spp.**	*Populus tremula* × *alba* shoot tips; *Populus trichocarpa* 1 single stem internode	snRNA-seq	FANS (FACS Aria IIU/III) from cell debris; 10x Genomics Chromium device	10x Genomics Single Cell 3′ Reagent Kit v3; Illumina platform	9430 (shoot); 7383 (stem rep1); 8245 (stem rep2)	Cell Ranger	2021	[34]
**rice**	aerial part of two-week-old rice seedlings	scRNA-seq	10x Genomics Chromium device	10x Genomics Single Cell 3′ Reagent Kit v2/3; Illumina platform	4580	Cell Ranger; STAR Aligner; UMAP; Scran; dbscan; UpSetR; Monocle; AgriGo	2020	[35]
	5 mm crown root tips				10968	Cell Ranger; t-SNE; Seurat; UMAP; Monocle; InParanoid	2021	[36]

Species	Organ	Sequencing method	Platform for single cell sorting	Platforms for cDNA libraries preparation and sequencing	Number of finally analyzed cells/nuclei	Bioinformatics soft and pipelines	Publication year	Reference
**rice**	1 cm radicles (from root tip) of wt rice	scRNA-seq	10x Genomics Chromium device	10x Genomics Single Cell 3′ Reagent Kit v2/3; Illumina platform	27469	Cell Ranger; STAR Aligner; DoubletFinder; Seurat; t-SNE; Scanpy; Velocyto CLI; ForceAtlas; Diffusion maps; AgriGO	2021	[37]
**tomato**	shoot borne root primordia at different developmental stages from *DR5:mScarleti-NLS TCSn:mNeonGreen-NLS* double transgenic line	scRNA-seq	FACS into 96-well plates (FACS Aria or Melody)	10x Genomics Single Cell 3′ Reagent Kit v2; Illumina platform	230/399/89/242 (cells from different stages)	STAR Aligner; zUMI pipeline; Seurat; CCA pipeline; Slingshot; UMAP; entropy R function	2020	[38]
	2 w old shoot apices (SAM together with the first three primordia)	snRNA-seq	10x Genomics Chromium device	10x Genomics Single Cell 3′ Reagent Kit v3; Illumina platform	13377	Cell Ranger; Seurat; Monocle; BLASTP; BEAM algorithm; Cytoscape; AgriGO; SCODE		[39]

Supplementary references

1. Brennecke, P.; Anders, S.; Kim, J.K.; Kołodziejczyk, A.A.; Zhang, X.; Proserpio, V.; Baying, B.; Benes, V.; Teichmann, S.A.; Marioni, J.C., et al. Accounting for technical noise in single-cell RNA-seq experiments. *Nature Methods* **2013**, *10*, 1093-1095, doi:10.1038/nmeth.2645.
2. Efroni, I.; Ip, P.-L.; Nawy, T.; Mello, A.; Birnbaum, K.D. Quantification of cell identity from single-cell gene expression profiles. *Genome Biology* **2015**, *16*, 1-12, doi:10.1186/s13059-015-0580-x.
3. Efroni, I.; Mello, A.; Nawy, T.; Ip, P.-L.; Rahni, R.; DelRose, N.; Powers, A.; Satija, R.; Birnbaum, K.D. Root regeneration triggers an embryo-like sequence guided by hormonal interactions. *Cell* **2016**, *165*, 1721-1733, doi:10.1016/j.cell.2016.04.046.
4. Denyer, T.; Ma, X.; Klesen, S.; Scacchi, E.; Nieselt, K.; Timmermans, M.C.P. Spatiotemporal developmental trajectories in the Arabidopsis root revealed using high-throughput single-cell RNA sequencing. *Developmental Cell* **2019**, *48*, 840-852, doi:10.1016/j.devcel.2019.02.022.
5. Jean-Baptiste, K.; McFaline-Figueroa, J.L.; Alexandre, C.M.; Dorrity, M.W.; Saunders, L.; Bubb, K.L.; Trapnell, C.; Fields, S.; Queitsch, C.; Cuperus, J.T. Dynamics of gene expression in single root cells of *Arabidopsis thaliana*. *The Plant Cell* **2019**, *31*, 993-1011, doi:10.1105/tpc.18.00785.
6. Ryu, K.H.; Huang, L.; Kang, H.M.; Schiefelbein, J. Single-cell RNA sequencing resolves molecular relationships among individual plant cells. *Plant Physiology* **2019**, *179*, 1444-1456, doi:10.1104/pp.18.01482.
7. Shulse, C.N.; Cole, B.J.; Ciobanu, D.; Lin, J.; Yoshinaga, Y.; Gouran, M.; Turco, G.M.; Zhu, Y.; O'Malley, R.C.; Brady, S.M., et al. High-throughput single-cell transcriptome profiling of plant cell types. *Cell Reports* **2019**, *27*, 2241-2247, doi:10.1016/j.celrep.2019.04.054.
8. Turco, G.M.; Rodriguez-Medina, J.; Siebert, S.; Han, D.; Valderrama-Gómez, M.Á.; Vahldick, H.; Shulse, C.N.; Cole, B.J.; Juliano, C.E.; Dickel, D.E., et al. Molecular mechanisms driving switch behavior in xylem cell differentiation. *Cell Reports* **2019**, *28*, 342-351, doi:10.1016/j.celrep.2019.06.041.
9. Zhang, T.-Q.; Xu, Z.-G.; Shang, G.-D.; Wang, J.-W. A single-cell RNA sequencing profiles the developmental landscape of *Arabidopsis* root. *Molecular Plant* **2019**, *12*, 648-660, doi:10.1016/j.molp.2019.04.004.
10. Coate, J.E.; Farmer, A.D.; Schiefelbein, J.W.; Doyle, J.J. Expression partitioning of duplicate genes at single cell resolution in Arabidopsis roots. *Frontiers in Genetics* **2020**, *11*, 1-22, doi:10.3389/fgene.2020.596150.
11. Gala, H.P.; Lanctot, A.P.; Jean-Baptiste, K.; Guiziou, S.; Chu, J.C.; Zemke, J.E.; George, W.; Queitsch, C.; Cuperus, J.; Nemhauser, J. A single cell view of the transcriptome during lateral root initiation in *Arabidopsis thaliana*. *bioRxiv* **2020**, 10.1101/2020.10.02.324327, doi:10.1101/2020.10.02.324327.
12. Liu, Z.; Zhou, Y.; Guo, J.; Li, J.; Tian, Z.; Zhu, Z.; Wang, J.; Wu, R.; Zhang, B.; Hu, Y., et al. Global dynamic molecular profiling of stomatal lineage cell development by single-cell RNA sequencing. *Molecular Plant* **2020**, *13*, 1178-1193, doi:10.1016/j.molp.2020.06.010.
13. Lopez-Anido, C.B.; Vatén, A.; Smoot, N.K.; Sharma, N.; Guo, V.; Gong, Y.; Anleu Gil, M.X.; Weimer, A.K.; Bergmann, D.C. Single-cell resolution of lineage trajectories in the Arabidopsis stomatal lineage and developing leaf. *bioRxiv* **2020**, 10.1101/2020.09.08.288498, doi:10.1101/2020.09.08.288498.
14. Picard, C.L.; Povilus, R.A.; Williams, B.P.; Gehring, M. Single nucleus analysis of Arabidopsis seeds reveals new cell types and imprinting dynamics. *bioRxiv* **2020**, 10.1101/2020.08.25.267476, doi:10.1101/2020.08.25.267476.

15. Shahan, R.; Hsu, C.-W.; Nolan, T.M.; Cole, B.J.; Taylor, I.W.; Vlot, A.H.C.; Benfey, P.N.; Ohler, U. A single cell Arabidopsis root atlas reveals developmental trajectories in wild type and cell identity mutants. *bioRxiv* **2020**, 10.1101/2020.06.29.178863, doi:10.1101/2020.06.29.178863.
16. Song, Q.; Ando, A.; Jiang, N.; Ikeda, Y.; Chen, Z.J. Single-cell RNA-seq analysis reveals ploidy-dependent and cell-specific transcriptome changes in Arabidopsis female gametophytes. *Genome Biology* **2020**, *21*, 1-18, doi:10.1186/s13059-020-02094-0.
17. Torii, K.; Kubota, A.; Araki, T.; Endo, M. Time-series single-cell RNA-seq data reveal auxin fluctuation during endocycle. *Plant and Cell Physiology* **2020**, *61*, 243-250, doi:10.1093/pcp/pcz228.
18. Wendrich, J.R.; Yang, B.; Vandamme, N.; Verstaen, K.; Smet, W.; Van de Velde, C.; Minne, M.; Wybouw, B.; Mor, E.; Arents, H.E., et al. Vascular transcription factors guide plant epidermal responses to limiting phosphate conditions. *Science* **2020**, *370*, 1-13 (first release), doi:10.1126/science.aay4970.
19. Dorrity, M.W.; Alexandre, C.M.; Hamm, M.; Vigil, A.-L.; Fields, S.; Queitsch, C.; Cuperus, J. The regulatory landscape of *Arabidopsis thaliana* roots at single-cell resolution. *bioRxiv* **2021**, 10.1101/2020.07.17.204792, doi:10.1101/2020.07.17.204792.
20. Farmer, A.; Thibivilliers, S.; Ryu, K.H.; Schiefelbein, J.; Libault, M. Single-nucleus RNA and ATAC sequencing reveals the impact of chromatin accessibility on gene expression in Arabidopsis roots at the single-cell level. *Molecular Plant* **2021**, *14*, 372-383, doi:10.1016/j.molp.2021.01.001.
21. Graeff, M.; Rana, S.; Wendrich, J.R.; Dorier, J.; Eekhout, T.; Aliaga Fandino, A.C.; Guex, N.; Bassel, G.W.; De Rybel, B.; Hardtke, C.S. A morpho-transcriptomic map of brassinosteroid action in the Arabidopsis root. *bioRxiv* **2021**, 10.1101/2021.03.30.437656, doi:10.1101/2021.03.30.437656.
22. Kao, P.; Schon, M.A.; Mosiolek, M.; Nodine, M.D. Gene expression variation in Arabidopsis embryos at single-nucleus resolution. *bioRxiv* **2021**, 10.1101/2021.03.26.437151, doi:10.1101/2021.03.26.437151.
23. Kim, J.-Y.; Symeonidi, E.; Pang, T.Y.; Denyer, T.; Weidauer, D.; Bezrutczyk, M.; Miras, M.; Zöllner, N.; Hartwig, T.; Wudick, M.M., et al. Distinct identities of leaf phloem cells revealed by single cell transcriptomics. *The Plant Cell* **2021**, *koaa060*, doi:10.1093/plcell/koaa060.
24. Long, Y.; Liu, Z.; Jia, J.; Mo, W.; Fang, L.; Lu, D.; Liu, B.; Zhang, H.; Chen, W.; Zhai, J. FlsnRNA-seq: protoplasting-free full-length single-nucleus RNA profiling in plants. *Genome Biology* **2021**, *22*, doi:10.1186/s13059-021-02288-0.
25. Roszak, P.; Heo, J.-o.; Blob, B.; Toyokura, K.; de Luis Balaguer, M.A.; Lau, W.W.Y.; Hamey, F.; Cirrone, J.; Wang, X.; Ursache, R., et al. Analysis of phloem trajectory links tissue maturation to cell specialization. *bioRxiv* **2021**, 10.1101/2021.01.18.427084, doi:10.1101/2021.01.18.427084.
26. Sunaga-Franze, D.Y.; Muino, J.M.; Braeuning, C.; Xu, X.; Zong, M.; Smaczniak, C.; Yan, W.; Fischer, C.; Vidal, R.; Kliem, M., et al. Single-nuclei RNA-sequencing of plant tissues. *bioRxiv* **2021**, 10.1101/2020.11.14.382812, doi:10.1101/2020.11.14.382812.
27. Zhang, T.-Q.; Chen, Y.; Wang, J.-W. A single-cell analysis of the Arabidopsis vegetative shoot apex. *Developmental Cell* **2021**, *56*, 1-19, doi:10.1016/j.devcel.2021.02.021.
28. Nelms, B.; Walbot, V. Defining the developmental program leading to meiosis in maize. *Science* **2019**, *364*, 52-56, doi:10.1126/science.aav6428.
29. Marand, A.P.; Chen, Z.; Gallavotti, A.; Schmitz, R.J. A cis-regulatory atlas in maize at single-cell resolution. *bioRxiv* **2020**, 10.1101/2020.09.27.315499, doi:10.1101/2020.09.27.315499.

30. Satterlee, J.W.; Strable, J.; Scanlon, M.J. Plant stem-cell organization and differentiation at single-cell resolution. *Proceedings of the National Academy of Sciences* **2020**, *117*, 33689-33699, doi:10.1073/pnas.2018788117.
31. Bezrutczyk, M.; Zöllner, N.R.; Kruse, C.P.S.; Hartwig, T.; Lautwein, T.; Köhrer, K.; Frommer, W.B.; Kim, J.-Y. Evidence for phloem loading via the abaxial bundle sheath cells in maize leaves. *The Plant Cell* **2021**, *koaa055*, doi:10.1093/plcell/koaa055.
32. Xu, X.; Crow, M.; Rice, B.R.; Li, F.; Harris, B.; Liu, L.; Demesa-Arevalo, E.; Lu, Z.; Wang, L.; Fox, N., et al. Single-cell RNA sequencing of developing maize ears facilitates functional analysis and trait candidate gene discovery. *Developmental Cell* **2021**, *56*, 557-568, doi:10.1016/j.devcel.2020.12.015.
33. Kubo, M.; Nishiyama, T.; Tamada, Y.; Sano, R.; Ishikawa, M.; Murata, T.; Imai, A.; Lang, D.; Demura, T.; Reski, R., et al. Single-cell transcriptome analysis of Physcomitrella leaf cells during reprogramming using microcapillary manipulation. *Nucleic Acids Research* **2019**, *47*, 4539-4553, doi:10.1093/nar/gkz181.
34. Conde, D.; Triozzi, P.M.; Balmant, K.M.; Doty, A.L.; Miranda, M.; Boullosa, A.; Schmidt, H.W.; Pereira, W.J.; Dervinis, C.; Kirst, M. A robust method of nuclei isolation for single-cell RNA sequencing of solid tissues from the plant genus Populus. *bioRxiv* **2021**, 10.1101/2021.02.11.430521, doi:10.1101/2021.02.11.430521.
35. Wang, Y.; Huan, Q.; Chu, X.; Li, K.; Qian, W. Single-cell transcriptome analyses recapitulate the cellular and developmental responses to abiotic stresses in rice. *bioRxiv* **2020**, 10.1101/2020.01.30.926329, doi:10.1101/2020.01.30.926329.
36. Liu, Q.; Liang, Z.; Feng, D.; Jiang, S.; Wang, Y.; Du, Z.; Li, R.; Hu, G.; Zhang, P.; Ma, Y., et al. Transcriptional landscape of rice roots at the single-cell resolution. *Molecular Plant* **2021**, *14*, 384-394, doi:10.1016/j.molp.2020.12.014.
37. Zhang, T.-Q.; Chen, Y.; Liu, Y.; Lin, W.-H.; Wang, J.-W. Single-cell transcriptome atlas and chromatin accessibility landscape reveal differentiation trajectories in the rice root. *Nature Communications* **2021**, *12*, 1-12, doi:10.1038/s41467-021-22352-4.
38. Omary, M.; Gil-Yarom, N.; Yahav, C.; Steiner, E.; Efroni, I. A conserved superlocus regulates above- and belowground root initiation. *bioRxiv* **2020**, 10.1101/2020.11.11.377937, doi:10.1101/2020.11.11.377937.
39. Tian, C.; Du, Q.; Xu, M.; Du, F.; Jiao, Y. Single-nucleus RNA-seq resolves spatiotemporal developmental trajectories in the tomato shoot apex. *bioRxiv* **2020**, 10.1101/2020.09.20.305029, doi:10.1101/2020.09.20.305029.
